# An integrated adaptive framework for mycotoxin management in African cereals: evidence from a scoping review

**DOI:** 10.1007/s12550-026-00673-x

**Published:** 2026-09-24

**Authors:** Vioutou E. C. Coffi, Kokeb Tesfamariam, Ogouyôm Herbert Iko Afé, Noël Akissoé, Sarah De Saeger, D. Sylvain Dabadé

**Affiliations:** 1 Laboratory of Food Science and Technology, University of Abomey-Calavi, 03 BP 2819, Jericho-Cotonou, Benin; 2 Centre of Excellence in Mycotoxicology and Public Health, Faculty of Pharmaceutical Sciences, Ghent University, Ghent, Belgium

**Keywords:** Mycotoxins, Cereals, Africa, Food safety

## Abstract

Mycotoxins threaten food safety, public health, and trade in Africa, where cereals are staple foods largely produced and consumed through informal systems. This scoping review synthesizes evidence on pre- and post-harvest mycotoxin management in African cereal systems and translates it into an integrated adaptive framework for policy and practice. A structured literature search conducted in Google Scholar, Web of Science, and Scopus identified 9,817 records, of which 33 primary studies met the eligibility criteria. The included studies covered interventions across the cereal value chain, including biocontrol, chemical pest management, drying, storage, and processing practices. Biocontrol interventions, particularly atoxigenic *Aspergillus flavus* products such as Aflasafe reduced aflatoxin levels by 60–100% across the reviewed studies, with long-term field evidence showing that more than 95% of treated grains remained below 20 µg/kg in Nigeria and 75% below 4 µg/kg in Senegal. Bt maize also reduced fumonisin contamination by approximately 74% in South Africa. Post-harvest interventions, including improved drying, hermetic storage, sorting, and HACCP-based processing, achieved variable but substantial reductions, with hand sorting reducing aflatoxin B1 and fumonisin B1 to less than 6% and 5% of initial levels, respectively, and HACCP-based processing reducing aflatoxins to below 5 µg/kg. However, persistent constraints, including climate variability, limited infrastructure, low awareness, gender inequities, informal market dominance, and fragmented regulatory frameworks, continue to limit large-scale effectiveness. These findings informed an Integrated Adaptive Mycotoxin Management Framework grounded in a One Health perspective to support sustainable mycotoxin risk reduction in African cereals.

**Supplementary information:**

The online version contains supplementary material available at https://doi.org/10.1007/s12550-026-00673-x.

## Introduction

Cereals are plants cultivated for their edible grains and are a major source of carbohydrates in the human diet (Laskowski et al. 2019). Cereals such as maize, sorghum, and millet, which constitute major staples across African regions, alongside other cereals such as wheat, rice, barley, oats, and rye play a crucial role in global food security (Grote et al. 2021). Among these, maize, or *Zea mays*, stands out for its versatility and significant contribution to human and animal feeding, as well as to various industries (Tanumihardjo et al. 2020). In low-income countries, particularly in Africa, maize is a staple food, with 54% of the continent’s maize production destined for human consumption and annual per capita consumption levels exceeding 50 kg (FAOSTAT, 2022). Cereals also play an important economic role as a source of income for farmers.

However, cereals are highly susceptible to contamination by mycotoxins, which are toxic substances produced by certain fungi (Khodaei et al. 2021). These fungi often develop under inappropriate storage conditions or during periods of drought or excessive moisture during crop growth (Dodds et al. 2020). According to Mousavi Khaneghah et al. (2019), the mycotoxins commonly associated with cereals include aflatoxins (AFs), fumonisins (FBs), ochratoxin A (OTA), deoxynivalenol (DON) and zearalenone (ZEN). These mycotoxins pose a serious threat to health, as the consumption of contaminated cereals is associated with a wide range of adverse effects, including gastrointestinal disorders, liver damage, increased cancer risk, partial immunosuppression, and adverse developmental outcomes such as birth defects, particularly in vulnerable populations (EFSA 2017).

The prevalence of mycotoxins in African cereals has been documented, raising major public health concerns (Kebede et al. 2020; Udomkun et al. 2017). This is especially concerning as children, with their developing immune systems, are particularly vulnerable to these toxins through their daily diet (Chilaka and Mally 2020). Mycotoxins have a significant socio-economic impact in Africa (Gbashi et al. 2019). Recent evidence indicates that 60–80% of food crops may contain detectable levels of at least one mycotoxin at some stage of the food chain, highlighting the pervasive nature of mycotoxin contamination in food systems (Eskola et al. 2020). These direct economic losses include reduction of the agricultural yields and the reduction in sales price of contaminated products. Furthermore, mycotoxin contamination of crops reduces the competitiveness of African agricultural products in international markets, leading to income losses for farmers and exporting countries (Udomkun et al. 2017). Kimanya (2015) found that the health risks of mycotoxin exposure in Africa are illustrated by the severe aflatoxicosis outbreaks in Kenya, with a 39.4% mortality rate in 2004 (125 deaths out of 317 cases) and a 44.2% mortality rate between 2005 and 2010 (46 deaths among 104 cases). Beyond acute aflatoxicosis outbreaks, chronic dietary exposure to aflatoxins represents a more pervasive and often underestimated public health burden in Africa. Long-term exposure has been associated with a range of non-communicable health outcomes, including hepatocellular carcinoma, immune suppression, and impaired child growth, particularly stunting (Chilaka and Mally 2020; Kebede et al. 2020). In addition, chronic exposure may contribute to micronutrient deficiencies and reduced nutrient absorption, further exacerbating malnutrition in vulnerable populations. These cumulative health effects highlight that the impact of mycotoxins extends beyond acute mortality and may result in substantial long-term human and socio-economic losses.

Given the critical importance of cereals in the human diet and the well-documented hazards posed by mycotoxins, which affect both developed and developing regions, effective management strategies are essential to safeguard public health, particularly in African contexts where exposure risks and structural constraints are often more pronounced. Previous reviews have largely focused on specific interventions, such as biocontrol or storage, or on regulatory aspects at global or regional levels. However, a comprehensive synthesis of primary evidence on how pre-harvest and post-harvest interventions perform across the cereal value chain in African contexts, and how contextual factors shape their effectiveness, remains limited.

Therefore, this scoping review aims to map and synthesize evidence from studies evaluating pre-harvest and post-harvest mycotoxin management interventions in African cereal systems, and to translate these findings into the Integrated Adaptive Mycotoxin Management Framework (IAMMF), proposed as a novel contribution of this study to inform context-specific policy and practice. Beyond mapping intervention effectiveness, this study adopts an integrative perspective that examines how technical, structural, and socio-economic factors interact to shape mycotoxin risk management outcomes in African cereal systems. By explicitly linking evidence on intervention performance with contextual constraints and enabling factors, the review provides the basis for developing a comprehensive and adaptive management framework that reflects real-world conditions across the value chain.

These findings indicate that isolated or single-stage interventions are often insufficient to ensure sustainable mycotoxin control. The variability in effectiveness observed across studies highlights that technical solutions alone are not sufficient, as their performance is strongly influenced by institutional, socio-economic, and environmental conditions. This underscores the need for integrated approaches that combine technical interventions with contextual and systemic factors, while remaining flexible to adapt to changing environmental and market conditions.

In this review, the One Health perspective is used as an integrative conceptual lens to situate mycotoxin management within the interconnected domains of human health, animal health, and environmental sustainability (Adisasmito et al. 2022). By integrating evidence across the cereal value chain and situating it within a systemic and adaptive framework, this study advances a structured approach that links prevention, monitoring, governance, and capacity building into a coherent management cycle tailored to African contexts.

In doing so, the review moves beyond intervention mapping and provides the conceptual foundation for an integrated and adaptive mycotoxin management strategy capable of responding to evolving risks and contextual constraints in African cereal systems.

## Methodology

This review focused specifically on cereal-based systems because cereals are major dietary staples across Africa and important vehicles for mycotoxin exposure. However, the exclusion of non-cereal commodities, particularly groundnuts, constitutes a limitation, as these commodities also contribute substantially to aflatoxin exposure in food and feed systems. Therefore, the One Health implications discussed here should be interpreted within the scope of cereal-based value chains rather than as a comprehensive assessment of all major aflatoxin exposure pathways in Africa. Future research should adopt a broader food-system approach that includes cereals and other high-risk commodities, such as groundnuts, to better capture implications for human health, animal health, and feed safety.

### Search strategy

A comprehensive literature search was conducted in Google Scholar, Web of Science, and Scopus using Boolean operators (Koumassa et al. 2025; Tohonon et al. 2024). The search strategy was designed to capture both broad mycotoxin-related studies and studies referring to specific toxins. Therefore, the search syntax combined generic terms related to mycotoxins with the names of major cereal-associated mycotoxins, including aflatoxins, fumonisins, ochratoxin A, deoxynivalenol, and zearalenone. The following search string was adapted to the requirements of each database:

(“Mycotoxins” OR “Mycotoxin contamination” OR “Aflatoxin” OR “Aflatoxins” OR “Fumonisin” OR “Fumonisins” OR “Ochratoxin A” OR “Deoxynivalenol” OR “DON” OR “Zearalenone” OR “ZEN”) AND (“Management” OR “Control” OR “Mitigation” OR “Prevention” OR “Reduction” OR “Intervention” OR “Biocontrol” OR “Biological control” OR “Good Agricultural Practices” OR “GAP” OR “Storage” OR “Processing” OR “Resistant varieties” OR “Decontamination”) AND (“Cereals” OR “Grains” OR “Maize” OR “Wheat” OR “Rice” OR “Sorghum” OR “Barley” OR “Millet”) AND (“Africa” OR “Sub-Saharan Africa” OR “North Africa” OR “East Africa” OR “West Africa” OR “Southern Africa”).

To increase coverage, the name of each African country, as defined by the African Union Commission (2022), was also combined with the core search terms. No restriction on publication year was applied.

## Eligibility criteria

Studies were eligible if they were conducted in Africa, published in English or French, and reported empirical evidence on pre-harvest and/or post-harvest interventions or practices aimed at preventing, reducing, or controlling mycotoxin contamination in cereals. Eligible studies included those reporting quantitative mycotoxin outcomes, such as prevalence, concentration, percentage reduction, or contamination levels before and after intervention or between treated and control groups. Studies were also considered when they assessed intervention-related risk factors directly linked to mycotoxin contamination, such as insect damage, fungal growth, mould occurrence, grain moisture, storage conditions, or grain quality. Studies were included irrespective of whether the intervention showed high, moderate, limited, or no effectiveness. Studies that only described general practices without any mycotoxin-related outcome, risk indicator, or relevance to contamination control were excluded.

## Study selection process

All identified records were exported into Mendeley Desktop 1.19.8, where duplicates were removed. A total of 9,817 records were retrieved and screened by the main author. To ensure methodological rigor and consistency, the screening process was supervised by senior researchers. Specifically, the supervisors independently reviewed a subset of titles, abstracts, and full-text articles to validate inclusion and exclusion decisions. Discrepancies were discussed and resolved through consensus. In addition, regular consultations were held throughout the screening process to ensure alignment with the eligibility criteria and to minimize selection bias.

Data extraction was conducted using a structured and standardized template to ensure consistency across studies. For each included study, the following information was systematically collected: country of study, cereal type, type of intervention (pre- or post-harvest), targeted mycotoxins, study design, and reported outcomes, including quantitative mycotoxin measures where available, such as concentration levels, percentage reduction, or prevalence. For studies without direct quantitative mycotoxin reduction data, intervention-related risk indicators were also extracted, including insect damage, fungal growth, mould occurrence, grain moisture, storage conditions, and grain quality. In addition, contextual information such as implementation conditions, adoption constraints, and reported limitations was also extracted when available to support the qualitative synthesis. The extraction process was performed by the main author and reviewed by senior researchers to ensure accuracy and consistency. This structured approach minimized subjectivity and enhanced the reproducibility of the review.

**Fig. 1:**
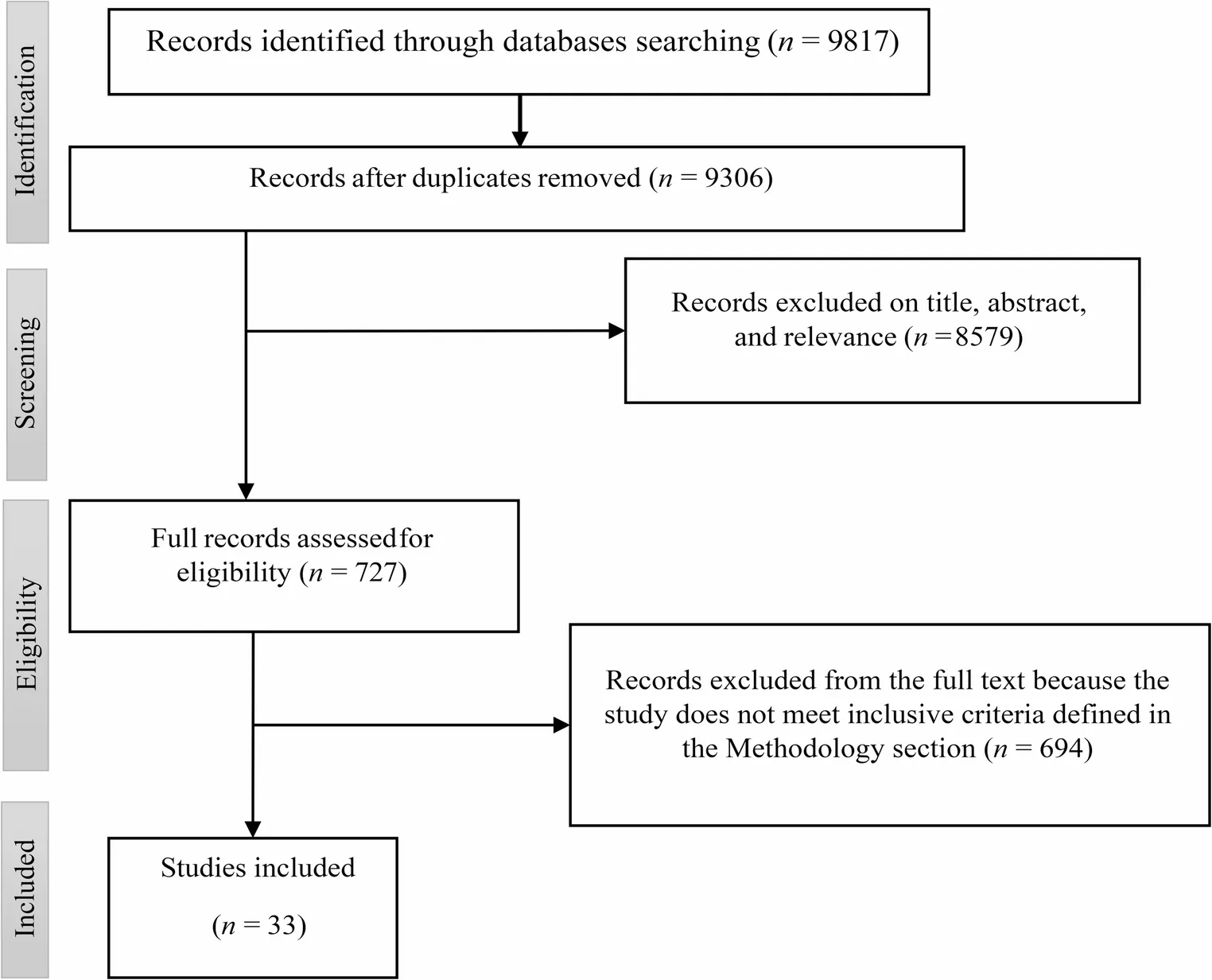
Flow chart of study selection

A total of 9,817 records were initially identified through database searching. After the removal of duplicates, 9,306 records remained for screening. Of these, 8,579 were excluded based on title and abstract screening. The remaining 727 full-text articles were assessed for eligibility, leading to the exclusion of 694 studies. Finally, 33 studies met the inclusion criteria and were included in the review. The study selection process is illustrated in Fig. 1. In addition to intervention-specific outcomes, the synthesis also captured cross-cutting implementation determinants such as gender dynamics and decision-support tools (e.g., predictive approaches) when they were reported within included studies.

## Data synthesis

The characteristics of included studies are summarized in Table 1.

**Table 1:** Characteristics of the 33 studies included in the review on mycotoxin management strategies in cereals in Africa

Intervention type	Country	Cereals	Key findings	References
Biocontrol (Aflasafe)	Nigeria	Maize	AFs reduction > 80%; high adoption rate (66.9%)	Johnson et al. (2018)
Biocontrol (Aflasafe)	Nigeria	Maize	Long-term efficacy; >95% of grains < 20 µg/kg	Bandyopadhyay et al. (2019)
Biocontrol (Aflasafe GH02)	Ghana	Maize, Groundnut	Reduced AFs 80–100% across AEZs	Agbetiameh et al. (2020)
Biocontrol (Aflasafe SN01)	Senegal	Maize	58–100% reduction; 75% <4 µg/kg	Senghor et al. (2020)
Biocontrol (Aflasafe SN01)	Gambia	Maize	82–100% AFs reduction in treated crops	Senghor et al. (2021)
Biocontrol (Aflasafe TZ01)	Tanzania	Maize	62% reduction AFB1 numerical increase in Fusarium toxins (not significant)	Fundikira et al. (2025)
Biocontrol (native atoxigenic *A. flavus* mixture)	Nigeria	Maize	AFs reductions of 67–95% at harvest	Atehnkeng et al. (2014)
Biocontrol (Aflasafe frequency of application)	Nigeria	Maize	AFs reductions at harvest ranged from 52.6–95.6%; repeated applications produced strong reductions, up to 95.6% at harvest and 98.1% after storage	Atehnkeng et al. (2022)
Biocontrol (Aflasafe TZ01 & TZ02)	Tanzania	Maize	Reduced AFs in maize; reported reduction > 85%	Mahuku et al. (2023)
Biocontrol (Aflasafe BF01 & SN01)	Burkina Faso, Mali, Niger, Togo	Maize, sorghum	Treated crops had > 80% less AFs than untreated crops	Bonkoungou et al. (2024)
Biocontrol (Aflasafe MWMZ01 & MZ02)	Mozambique	Maize	AFs reductions ranged from 61–93% in maize	Augusto et al. (2024)
Bt maize (insect-mediated fumonisin reduction)	South Africa	Maize	FBs was about 74% lower in Bt control maize than non-Bt control maize	Ncube et al. (2018)
Pesticides	Benin	Rice, Maize	No quantitative reduction reported; indirect effect on mycotoxin risk through pest control; environmental and health risks	Ahouangninou et al. (2012)
Pesticides	Benin	Rice	No quantitative reduction reported; pesticide use targeting insect pests rather than mycotoxins	Toffa et al. (2021)
Pesticides/Storage	Kenya	Maize	No quantitative reduction reported; combined pesticide use and storage practices associated with variable contamination levels	Midega et al. (2016)
Sun drying	Kenya	Maize	No quantitative reduction reported; traditional drying practice associated with continued contamination risks	De Groote et al. (2021)
Sun drying	Ghana	Maize	No quantitative reduction reported; widespread traditional practice with limited mycotoxin control effectiveness	Akowuah et al. (2018)
Field drying	Tanzania	Maize	No quantitative reduction reported; field drying and pre-storage practices influence grain quality and mycotoxin occurrence	Mutungi et al. (2019)
Suspended drying	Benin	Sorghum, Millet	No quantitative reduction reported; suspended drying reduces mould growth and may indirectly limit mycotoxin development	Tovide et al. (2017)
Storage structures	Ghana	Maize	No quantitative reduction reported; use of cribs and warehouses associated with insect infestation and residual contamination	Danso et al. (2019)
Storage resilience	Zimbabwe	Maize	No quantitative reduction reported; traditional storage structures vulnerable to climate variability and contamination risks	Mapfeka et al. (2019)
Rhombus storage	Nigeria	Maize, Sorghum	No quantitative reduction reported; rhombus storage effective for grain preservation but used by a minority of farmers	Okparavero et al. (2024)
Rhombus storage	Ethiopia	Sorghum	No quantitative reduction reported; traditional rhombus storage associated with effective grain preservation	Hussen (2021)
Granary storage	Senegal	Maize	No quantitative reduction reported; granary storage widely used, with variable effectiveness in limiting contamination	Gueye et al. (2013)
Storage (Rhombus, bags, cribs)	Nigeria	Sorghum	No quantitative reduction reported; survey data indicate continued reliance on traditional storage methods with variable contamination control	Mahai et al. (2015)
Processing (sorting, washing, dehulling)	Malawi	Maize	Quantitative reduction reported; hand sorting reduced AFB1 to < 6% and FB1 to < 5% of initial levels	Matumba et al. (2015)
Processing (HACCP)	Tanzania	Maize	Quantitative reduction reported; HACCP processing achieved AF < 5 µg/kg and FB < 2 µg/g in final flours	Ngure et al. (2024)
Traditional processing	Nigeria	Sorghum, Millet	No quantitative reduction reported; traditional sorting, cleaning, and fermentation practices associated with lower contamination risk	Egwim et al. (2013)
Traditional processing	Nigeria	Sorghum, Millet	No quantitative reduction reported; traditional cereal fermentation associated with improved food safety and potential reduction of mycotoxin risk through lactic acid bacteria activity	Achi and Asamudo (2019)
Traditional processing into *mawè*, *makume*, *akassa* and *owo*	Benin	Maize	*Makume*: reduction of AFs (93%) FBs (87%) *Akassa*: reduction AFs (92%), FBs (50%) *Owo*: reduction AFs (40%), FBs (48%)	Fandohan et al. (2005)
Hand sorting and washing	South Africa	Maize	FBs reduction of 84%	van der Westhuizen et al. (2010)
Traditional beverage processing	Nigeria	Maize, sorghum	Mycotoxins reduced by 76.2–99.9% in *kunu-zaki* and 59.3–94.8% in *pito*	Ezekiel et al. (2015)
Lactic acid fermentation (commercial *ogi* processing)	Nigeria	Maize	Reduction to the acceptable limit	Ademola et al. (2021)

The relatively limited number of included studies reflects the scarcity of primary field-based intervention studies in African cereal systems that assess mycotoxin mitigation outcomes or directly related contamination risk indicators, rather than a restrictive selection process. While the broader African mycotoxin literature is extensive, a large proportion of studies focus on occurrence, exposure assessment, toxicology, or general management practices without evaluating intervention effectiveness or mycotoxin-related risk indicators. In addition to extracting intervention outcomes and mycotoxin-related risk indicators, the synthesis also identified recurrent cross-cutting themes reported across included studies, including infrastructural constraints, governance challenges, gender-related factors, awareness gaps, and adaptive management needs. These themes were subsequently integrated through an interpretative synthesis approach to contextualize intervention effectiveness and inform the development of the Integrated Adaptive Mycotoxin Management Framework (IAMMF). To strengthen the One Health interpretation, a complementary targeted literature consultation was conducted after the intervention synthesis. This consultation was not intended to redefine the main corpus of included intervention studies or to constitute a separate systematic review, but rather to support the conceptual interpretation of potential co-benefits across human, animal, and environmental health domains. The targeted keywords were used iteratively in combinations linking the intervention categories identified in the review with relevant One Health dimensions. Then, intervention-related terms such as “biocontrol”, “Aflasafe”, “Bt maize”, “pesticide use”, “drying”, “storage”, “hermetic storage”, and “processing” were combined with terms such as “One Health”, “animal health”, “feed safety”, “animal feed”, “livestock”, “environmental health”, “fungal ecology”, “pesticide residues”, “grain spoilage”, “post-harvest losses”, “contaminated grain”, “by-products”, and “waste management”. Relevant sources were used narratively to support the One Health mapping and interpretation of potential co-benefits, without altering the final set of included intervention studies. Some studies already included in the main intervention synthesis were also relevant to the complementary One Health interpretation when they reported information related to feed safety, fungal ecology, post-harvest losses, pesticide residues, contaminated by-products, or environmental considerations. These studies were not counted as additional records, but were used alongside complementary sources to support the interpretive mapping of potential One Health co-benefits.

## Results and discussion

This section presents the main interventions identified across the literature, together with their reported outcomes and interpretation, in order to establish a direct link between the evidence generated and its practical implications. Mycotoxin management in cereals requires an integrated approach that connects food safety, public health, agricultural productivity, animal health, and environmental sustainability. In line with the One Health paradigm, the results presented in this section examine how interventions implemented at different stages of the cereal value chain can generate co-benefits across human, animal, and environmental health domains, while taking into account the socio-economic realities of African cereal systems.

In addition to the intervention outcomes reported in the included studies, complementary literature identified through the targeted One Health search was used to support the interpretation of these findings within a One Health perspective. This allowed the interventions identified across the cereal value chain to be discussed not only in terms of their effects on mycotoxin reduction, but also in relation to their potential co-benefits for human health, animal health, and environmental sustainability. Although extensive literature exists on mycotoxin occurrence and exposure in Africa, relatively few studies have empirically evaluated intervention effectiveness or directly related contamination risk indicators across pre- and post-harvest stages of the cereal value chain, highlighting an important evidence gap for future research.

### Mycotoxin management strategies in cereals in Africa

Mycotoxin management strategies in Africa are based on interventions at every stage of the food supply chain, from farm to fork (Ndemera et al. 2020). There are pre-harvest strategies and post-harvest strategies.

### Pre-harvest strategies

Based on the included studies, pre-harvest strategies reported in the African context primarily focused on biocontrol approaches and chemical pest management. Other commonly recommended pre-harvest practices, such as crop rotation, resistant varieties, and Good Agricultural Practices (GAP), were rarely evaluated as standalone mycotoxin mitigation interventions in the eligible studies and are therefore discussed as gaps rather than results.

### Use of biocontrol agents

Biocontrol agents, such as *Aflasafe* and Bt maize, play a crucial role in reducing mycotoxin levels, particularly AFs and FBs. *Aflasafe* consists of non-toxigenic strains of *Aspergillus flavus* that, when applied to fields, competitively exclude toxigenic strains naturally present in the soil. Among the 33 studies reviewed, 11 focused on biocontrol interventions.

These studies consistently reported substantial AFs reductions ranging from 60% to 100% across diverse agroecological conditions in Nigeria, Ghana, Senegal, the Gambia, and Tanzania. For instance, Bandyopadhyay et al. (2019) demonstrated sustained efficacy over a decade of field trials, with over 90% of maize samples below international AFs limits, while Johnson et al. (2018) observed strong adoption among Nigerian smallholders integrating *Aflasafe* with other agricultural inputs. Field applications also revealed a shift in fungal populations towards non-aflatoxigenic *A. flavus* strains, confirming the ecological success of the biocontrol (Agbetiameh et al. 2020; Senghor et al. 2020, 2021). Similarly, additional field studies further confirmed the effectiveness of *Aflasafe*-based biocontrol interventions across African cereal systems. Reported AF reductions ranged from 52.6% to 98.1% in Nigeria (Atehnkeng et al. 2014, 2022), exceeded 85% in Tanzania (Mahuku et al. 2023), and were greater than 80% in maize and sorghum systems in Burkina Faso, Mali, Niger, and Togo (Bonkoungou et al. 2024). AF reductions ranging from 61% to 93% were also reported in maize systems in Mozambique (Augusto et al. 2024). These findings further demonstrate the effectiveness of *Aflasafe* across diverse agroecological conditions in Africa.

From an exposure-reduction perspective, these effects are likely to be meaningful because several studies reported not only relative AF reductions, but also reductions to levels below commonly used regulatory or acceptability thresholds. For example, long-term field evidence from Nigeria reported that more than 95% of grains from treated fields contained AF levels below 20 µg/kg (Bandyopadhyay et al. 2019), while studies in Senegal reported that 75% of treated maize samples were below 4 µg/kg (Senghor et al. 2020). Such findings suggest that *Aflasafe*-based biocontrol can contribute not only to lowering contamination, but also to increasing the proportion of cereal lots suitable for safer food and feed use.

Despite its proven effectiveness, large-scale deployment remains limited by climatic variability, regulatory disparities, costs, and uneven commercialization across countries. Moreover, *Aflasafe* targets AFs specifically and may not control co-occurring toxins such as FBs, highlighting the need for integrated management strategies (Fundikira et al. 2025).

In addition, genetically modified Bt maize has been shown to reduce insect damage, thereby limiting Fusarium infection and subsequent FBs contamination (Rheeder and van der Westhuizen 2024). In the South African study included in this review, fumonisin concentrations were approximately 74% lower in Bt maize than in the non-Bt control, indicating a substantial indirect effect on FBs contamination through reduced insect-mediated fungal infection (Ncube et al. 2018). However, its adoption remains limited in many African countries due to regulatory, economic, infrastructural and societal factors (Gbadegesin et al. 2022). Although Bt maize represents a potentially effective pre-harvest strategy for reducing FBs-related contamination risks, the current African evidence base remains geographically concentrated, mainly in South Africa, and its effectiveness under diverse smallholder and multi-mycotoxin contexts requires further investigation (Rheeder and van der Westhuizen 2024). This reduction may be relevant from an exposure perspective in settings where maize is consumed frequently and contributes substantially to FBs intake. However, because the available African evidence remains geographically limited and does not include dietary exposure modelling or health outcome assessment, its public health significance should be interpreted as potential exposure reduction rather than demonstrated clinical benefit (Rheeder and van der Westhuizen 2024).

Interpreted through a One Health lens, and supported by the complementary One Health literature used to frame this review, biocontrol-based interventions generate potential co-benefits beyond mycotoxin reduction alone (Adisasmito et al. 2022). For human health, reductions in AFs and FBs may contribute to lowering dietary exposure in populations highly dependent on maize and sorghum, particularly where treated grains fall below regulatory or acceptability thresholds (Bandyopadhyay et al. 2019; Ncube et al. 2018; Rheeder and van der Westhuizen 2024; Senghor et al. 2020). For animal health, safer cereal grains may reduce the use of contaminated feed and support livestock productivity and the safety of animal-derived foods. From an environmental perspective, *Aflasafe* represents an ecologically based strategy that shifts fungal populations towards non-aflatoxigenic strains, while Bt maize may reduce insect-mediated fungal infection but requires careful biosafety, resistance-management, and regulatory oversight (Bryden 2012; Nwaji et al. 2022; Yang et al. 2020). These interventions therefore illustrate the relevance of integrated mycotoxin management, but their One Health benefits depend on context-specific implementation, farmer access, regulatory capacity, and long-term monitoring (Rheeder and van der Westhuizen 2024).

### Use of pesticides

The use of pesticides remains common across African cereal systems to control insect pests and fungal pathogens. By reducing pest damage and fungal infections that facilitate colonization by toxigenic species such as *Aspergillus* and *Fusarium*, pesticides can indirectly limit mycotoxin formation (Midega et al. 2016). Among the 33 studies reviewed, three investigated pesticides use in cereals.

These studies reported that various insecticides and fungicides were routinely applied in countries such as Benin and Kenya, including carbofuran, chlorpyrifos-ethyl, lambda-cyhalothrin, and mancozeb (Ahouangninou et al. 2012; Midega et al. 2016; Toffa et al. 2021). In some contexts, locally formulated products such as *Piapia* (based on dichlorvos) were also used for pest control. Although pesticide application can reduce insect-induced entry points for fungal infection, its contribution to overall mycotoxin mitigation remains limited. Pesticides are nevertheless reported here because they were explicitly evaluated in several included studies as part of pre-harvest management practices, reflecting their widespread use in African cereal systems rather than their effectiveness as primary mycotoxin control measures.

Persistent challenges include the lack of training on safe handling, inappropriate application methods, and the absence of monitoring systems for pesticide residues in food (Adeyeye et al. 2022). In addition, pesticides cannot neutralize toxins once produced, and repeated use may lead to chemical accumulation in soils and crops, creating secondary food safety concerns (Adeyeye et al. 2022; Liu et al. 2022).

These findings underscore the need for integrated pest and mycotoxin management approaches that combine biological, cultural, and chemical strategies within safer and more sustainable frameworks. Within this One Health-informed interpretation, pesticide use should be considered as a risk–trade-off intervention rather than a standalone mycotoxin control strategy. By reducing insect damage, pesticide-based pest control may indirectly limit fungal entry points and subsequent mycotoxin formation (Beyuo et al. 2024). However, these potential benefits must be balanced against concerns related to pesticide residues, inappropriate use, environmental contamination, and effects on non-target organisms (Adeyeye et al. 2022). Therefore, pesticide use can contribute to integrated mycotoxin management only when supported by appropriate farmer training, residue monitoring, regulatory oversight, and safer application practices (Nyarko et al. 2021).

### Post-harvest strategies

Post-harvest practices play a crucial role in mycotoxin management, particularly in cereals, where contamination can continue after harvest if conditions are not controlled (Neme and Mohammed 2017). Among these practices, proper drying and storage are essential.

### Drying

Drying is one of the most common and critical post-harvest practices for preventing fungal growth and mycotoxin formation in cereals (Nwaigwe 2019). Among the 33 studies included in this review, four addressed drying techniques across different African contexts. These studies consistently highlighted that effective moisture reduction is key to limiting fungal proliferation and toxin production.

Traditional open-air sun drying remains predominant among smallholder farmers in several African countries and was therefore frequently reported in the included studies, despite being widely recognised as a suboptimal practice for mycotoxin prevention (Akowuah et al. 2018; De Groote et al. 2021; Mutungi et al. 2019; Tovide et al. 2017). While it is a low-cost and accessible method, this practice exposes grains to soil contamination, dust, pests, and fluctuating weather conditions, particularly in humid regions. In addition, leaving grains to dry overnight may expose them to rehydration due to dew or unexpected rainfall, as well as to risks of theft or animal intrusion, further compromising drying effectiveness (Matavel et al. 2021). Similarly, the adoption of improved drying technologies, such as solar or hybrid dryers, may be hindered by concerns related to equipment security and maintenance, including the potential loss or damage of key components such as panels, batteries, or ventilation systems (Udomkun et al. 2020). Some farmers adopt improved techniques such as raised or suspended drying structures, which enhance ventilation and reduce mold development, though their adoption remains limited and largely informal, as these structures are often farmer-built, lack standardized designs, and are rarely supported by extension services or formal quality control mechanisms (Udomkun et al. 2020).

Overall, drying interventions can substantially reduce the risk of mycotoxin contamination by maintaining grain moisture below safe thresholds; typically below 13–14% as recommended in many developed systems to limit fungal growth and toxin production (Chulze 2010). However, their effectiveness depends on local climatic conditions, drying infrastructure, and farmers’ technical capacity. Promoting improved and weather-resilient drying technologies, including solar or hybrid dryers, could enhance mycotoxin prevention while ensuring better product quality and marketability. From a One Health-informed perspective, improved drying represents a primary prevention strategy in cereal mycotoxin management (Nyarko et al. 2021). By rapidly reducing grain moisture after harvest, drying interventions may limit fungal growth and toxin accumulation, thereby helping to preserve safer cereals for household consumption and animal feed (Neme and Mohammed 2017). Their broader relevance also lies in reducing spoilage and post-harvest losses, although their impact depends on climatic conditions, access to appropriate drying infrastructure, and farmer adoption (Udomkun et al. 2020).

### Storage

Storage is a decisive stage in mycotoxin management, as poor post-harvest conditions can favor the growth of *Aspergillus* and *Fusarium* species, leading to toxin accumulation (Mobolade et al. 2019). Seven of the 33 studies reviewed addressed storage practices, revealing a diversity of traditional and improved systems across Africa.

Traditional methods, including granaries made of earth, palm leaves, or thatch, as well as cribs and rhombus structures, remained the most widespread among smallholder farmers in countries such as Benin, Ghana, Nigeria, Ethiopia, Senegal, and Zimbabwe (Danso et al. 2019; Gueye et al. 2013; Hussen 2021; Mapfeka et al. 2019; Tovide et al. 2017). Although these systems are low-cost and locally adapted, they are rarely airtight and therefore prone to insect infestations, rodent attacks, and cross-contamination between grain batches. Their effectiveness in preventing fungal development and toxin accumulation varies considerably depending on material durability and maintenance.

In some regions, particularly in Ethiopia, underground pit storage systems are also traditionally used for cereal preservation (Hussen 2021). While these systems may provide protection against pests and environmental exposure, they can create conditions of elevated moisture and limited ventilation, potentially increasing the risk of fungal growth and mycotoxin development if not properly managed (Hussen 2021).

In contrast, hermetic storage technologies such as metal silos, Purdue Improved Crop Storage (PICS) bags, and improved warehouses have shown higher efficiency in reducing moisture, insect activity, and mycotoxin build-up (Opoku et al. 2023). However, their large-scale adoption remains limited by cost, availability, and farmers’ technical awareness (Baoua et al. 2016). Improved and hermetic storage contributes to a One Health-informed management pathway by preserving grain quality after harvest and limiting conditions that favour insect activity, moisture exchange, fungal proliferation, and further mycotoxin accumulation (Mobolade et al. 2019). These effects may protect household food stocks and reduce contamination in cereal-based feed materials (Nyarko et al. 2021). In addition, effective storage can help reduce post-harvest losses and may limit reliance on repeated chemical treatments during storage, provided that storage technologies are affordable, available, and correctly used (Baoua et al. 2016; Opoku et al. 2023).

Overall, improving grain storage in Africa requires combining traditional knowledge with affordable innovations that ensure better sealing and environmental control. Capacity building, cooperative investment, and local manufacturing of hermetic equipment could strengthen sustainable mycotoxin prevention at the household and community levels.

### Processing

Processing represents the final opportunity to reduce mycotoxin levels in cereals before consumption. Eight of the 33 studies reviewed examined processing interventions such as sorting, washing, dehulling, fermentation, and the application of HACCP-based frameworks (Achi and Asamudo 2019; Egwim et al., 2013; Matumba et al. 2015; Ngure et al. 2024).

Physical separation methods, including manual sorting, flotation, and dehulling, were shown to remove visibly contaminated grains and achieve marked reductions in mycotoxin concentrations. In Malawi, hand sorting reduced AFB1 to less than 6% and FB1 to less than 5% of their initial levels in contaminated maize, indicating reductions of more than 90% for both toxins (Matumba et al. 2015). Traditional cleaning and fermentation practices, as used in the preparation of local foods such as *Ogi*, *Agidi*, and *Fura*, also contributed to lowering fungal load and toxin exposure, though the degree of reduction depended on process hygiene and duration (Achi and Asamudo 2019; Egwim et al., 2013).

Additional evidence from African cereal processing systems further demonstrated the effectiveness of traditional processing practices in reducing mycotoxin contamination. In Benin, the processing of maize into local foods such as *mawè*, *makume*, *ogi*, *akassa*, and *owo* resulted in AFs reductions of up to 93% and fumonisin reductions of up to 87%, depending on the product and processing method (Fandohan et al. 2005). Similarly, hand sorting and washing of maize in South Africa achieved fumonisin reductions of approximately 84% (van der Westhuizen et al. 2010). In Nigeria, traditional beverage processing reduced mycotoxin levels by 76.2–99.9% in kunu-zaki and 59.3–94.8% in pito (Ezekiel et al. 2015), while lactic acid fermentation and commercial ogi processing reduced contamination to acceptable limits (Ademola et al. 2021). These processing-related reductions are particularly relevant from a public health perspective because they occur close to the point of consumption and can therefore directly lower the number of mycotoxins ingested from cereal-based foods. This is especially important for traditional cereal-based foods and beverages that are consumed frequently in many African settings (Tanumihardjo et al. 2020). However, the extent of exposure reduction depends on whether contaminated fractions are effectively discarded rather than reused for food or feed, and whether processing practices are hygienic, standardized, and consistently applied (Fandohan et al. 2005).

At an industrial level, the implementation of HACCP systems in milling and processing environments provided structured control of contamination points, leading to more consistent risk reduction (Ngure et al. 2024). In the Tanzanian HACCP-based processing study, final complementary flours achieved AF levels below 5 µg/kg and FB levels below 2 µg/g, suggesting that structured processing controls can reduce contamination to levels compatible with food safety targets. This provides stronger evidence of likely public health relevance than interventions that only reduce general contamination risk factors without measuring final toxin concentrations. However, widespread application is hindered by limited infrastructure, regulatory enforcement, and technical expertise (Chilaka et al. 2022). Processing interventions are particularly relevant within a One Health-informed framework because they act at the final exposure-reduction stage before consumption. Sorting, washing, dehulling, fermentation, and HACCP-based practices may reduce the amount of contaminated material entering human diets, while also influencing the safety of cereal by-products used as animal feed (Karlovsky et al. 2016). However, the environmental dimension depends on how rejected or contaminated fractions are managed, since unsafe disposal or reuse may transfer risks elsewhere in the food and feed chain (Chilaka et al. 2022).

Overall, the reviewed interventions demonstrated measurable reductions in mycotoxin contamination across multiple stages of the cereal value chain. Although most studies did not directly assess health outcomes, reductions below regulatory thresholds and substantial decreases relative to baseline levels suggest potential benefits for reducing chronic dietary exposure in highly cereal-dependent populations.

The synthesis of the included studies indicates that no single intervention can be considered universally sufficient to ensure sustained mycotoxin control across African cereal systems, particularly when considering multiple mycotoxins, diverse cereal value chains, post-harvest contamination risks, and adoption constraints. While biocontrol approaches demonstrated the highest and most consistent reductions in aflatoxins (60–100%), their evidence base remains mainly toxin-specific, and their long-term impact depends on adoption, correct application, regulatory support, and integration with complementary post-harvest and processing interventions. Post-harvest interventions, including drying, storage, and processing, showed variable effectiveness highly dependent on environmental conditions, infrastructure, and user practices. Notably, several widely promoted practices lacked robust quantitative evidence within the eligible studies, highlighting a gap between recommended practices and empirically validated interventions. These findings suggest that the effectiveness of mycotoxin mitigation strategies is strongly context-dependent and requires coordinated, multi-stage approaches rather than isolated interventions.

Beyond the magnitude of reduction reported across studies, the public health relevance of these interventions depends on their ability to reduce mycotoxin exposure below risk thresholds. While several interventions, particularly biocontrol and improved post-harvest practices, achieved substantial reductions in contamination levels, their translation into meaningful health benefits remains context-dependent. In settings where baseline contamination levels are extremely high, even partial reductions may significantly lower exposure and associated health risks. However, in the absence of integrated strategies and consistent adoption, these reductions may not be sufficient to ensure exposure levels below regulatory or health-based guidance values. This highlights the importance of combining interventions across the value chain to achieve clinically relevant risk reduction.

These findings also reveal that the effectiveness of technical interventions is systematically shaped by broader socio-economic, institutional, and behavioural factors influencing their adoption and scalability. This interdependence between technical performance and contextual constraints underscores the need for integrated management approaches that combine both technical and non-technical dimensions.

These limitations in effectiveness and scalability directly reflect broader structural, socio-economic, and institutional constraints that shape the implementation of mycotoxin management strategies in African cereal systems. These constraints are examined in the following section.

While the scoping review methodology was applied to identify and synthesise evidence on technical interventions, the following sections on challenges and cross-cutting dimensions are presented as analytical extensions derived from the patterns and limitations observed across the included studies. These sections aim to contextualise intervention effectiveness within broader structural, socio-economic, and institutional realities, rather than to provide a separate systematic review of these dimensions.

### Challenges to effective mycotoxin management in cereals in Africa

Several climatic, infrastructural, and socio-economic factors continue to constrain the effectiveness of mycotoxin management strategies across Africa. These challenges interact at multiple levels of the cereal value chain, from production and storage to processing and marketing, thereby reducing the impact of even the most effective interventions.

### Climatic and environmental drivers of mycotoxin proliferation in Africa

Climatic and environmental conditions play a decisive role in the occurrence of mycotoxins in African cereals. Hot and humid environments favour the growth of *Aspergillus* and *Fusarium* species, making contamination almost unavoidable (Fapohunda and Adewunmi 2019; Ngum et al. 2022). Climate variability particularly changes in temperature, rainfall, and relative humidity, further influenced fungal ecology and toxin production (Medina et al. 2014; Molnár et al. 2023). Rising temperatures and prolonged droughts weakened plant defences, promoted colonisation by toxigenic fungi, and concentrated toxins in grains (Knox et al. 2012; Sultan et al. 2013). Conversely, excessive rainfall and flooding increased post-harvest spoilage and mould growth (Magombeyi and Taigbenu 2008; Suleiman et al. 2017).

Regional differences illustrate these dynamics. In West Africa, recurrent droughts and erratic rainfall in the Sahel have increased AFs contamination, particularly in maize, millet, and sorghum (Gbashi et al. 2019; Roudier et al. 2011). In East Africa, heavy rains and limited storage facilities contributed to higher FBs and AFs levels (Kamala et al. 2015; Magembe et al. 2016), while in Southern Africa, alternating dry and wet periods exacerbated fungal proliferation and food security risks (Hove et al. 2016; Mboya and Kolanisi 2014).

Climate change also drives the geographic expansion of toxigenic fungi, exposing new areas and crops to contamination. Shifts in maize cultivation to higher altitudes, for instance, have not reduced *Aspergillus* infection risks (Kagot et al. 2019; Muthomi et al. 2012). Overall, climate change acts as a cross-cutting stressor that amplifies both pre- and post-harvest contamination risks, demanding adaptive management strategies tailored to local agroecological conditions.

### Infrastructural deficiencies and limited awareness

The effectiveness of mycotoxin management in Africa is severely constrained by weak infrastructure and limited technical capacity. Inadequate storage facilities contribute to grain deterioration and fungal proliferation, particularly in humid regions (Lehmane et al. 2022; Magembe et al. 2016). At the institutional level, insufficient laboratory capacity, limited access to advanced diagnostic technologies, and lack of coordinated surveillance systems restrict effective monitoring and enforcement (Chilaka et al. 2022). These structural weaknesses hinder early detection and response, leaving contaminated products to circulate in local markets.

Beyond technical limitations, low awareness among farmers, traders, and consumers further exacerbates the problem. Many smallholders remain unaware of the links between visible mould, moisture, and toxin formation, leading to inadequate drying and storage practices (Mboya and Kolanisi 2014). Even when knowledge improves, financial constraints and competing livelihood priorities often prevent behavioural change (Anitha et al. 2019). As a result, unsafe practices persist, such as the consumption or sale of visibly damaged grains.

The fragmentation of food supply chains and inconsistent regulatory enforcement compound these challenges. Mycotoxin regulations, where they exist, are often poorly implemented due to limited institutional coordination and scarce resources (Matumba et al. 2017; Senghor et al. 2021). Meanwhile, excessive reliance on chemical treatments without proper oversight can lead to residue accumulation and additional food safety risks (Gueye et al. 2020). Addressing these constraints requires integrated actions that combine infrastructure investment, capacity building, and sustained awareness campaigns targeting both producers and consumers.

### Gender disparities in mycotoxin management

Gender-specific challenges in managing mycotoxins in African cereals are deeply intertwined with broader socio-economic and cultural dynamics that directly affect food safety and public health (Murage et al. 2015). Indeed, in many rural communities, women are the primary agents in harvesting, storing, and processing cereals, yet they often operate under significant constraints that compromise their ability to effectively manage mycotoxin risks (Ezekiel et al. 2018). Firstly, limited access to financial resources restricts women from investing in modern storage facilities and drying technologies that are crucial to preventing fungal proliferation (El-shal and Morsy 2020). This economic barrier forces many to rely on traditional methods, which may be inadequate in mitigating contamination risks. Moreover, according to Cervini et al. (2023), systemic gaps in education and targeted training further exacerbate this vulnerability. Without sufficient technical support, women are less likely to adopt preventive measures or recognize early signs of contamination, thereby increasing the likelihood of mycotoxin exposure in household food supplies. In addition to economic and educational constraints, cultural and social structures frequently marginalize women in decision-making processes related to agricultural practices and food safety policies (Akanle et al., 2017). Despite being central to the agricultural value chain, women’s voices are often underrepresented in forums where strategies are devised and implemented (Wambui and Marther 2018). This exclusion not only limits the dissemination of critical information on mycotoxin hazards but also stifles the integration of gender-specific insights that could lead to more effective and contextually relevant control measures (Akanle et al., 2017).

### Variability in mycotoxin standards for mycotoxins regulatory framework

The regulation of mycotoxins plays a key role in protecting public health and food safety, but it still varies widely from one country to another (Egmond and Jonker 2003). Indeed, an analysis of the maximum mycotoxin limits set by various African countries (Table S1) reveals a marked heterogeneity in regulations. For the same type of product, regulatory thresholds can vary considerably. For example, the maximum authorized level of AFB1 in food products is 2 µg/kg in Tunisia, while it is set at 20 µg/kg in Nigeria and Kenya. Similarly, OTA is regulated at 3 µg/kg in Benin for certain cereal products, but can reach 30 µg/kg in Morocco. Some countries apply strict standards covering several types of mycotoxins and a wide range of products, while others focus mainly on AFB1. In addition, several nations do not yet have well-defined regulations on certain categories.

Moreover, reported data for raw maize across several African countries (Table S2) show substantial variability in mycotoxin occurrence and concentration levels, with some studies reporting very high values. Where available, mean or median concentrations are presented alongside maxima, which reflect high-contamination events rather than typical exposure levels. In Algeria, FBs were prominent, with FB1 detected in 96.6% of maize samples at a mean of 14,812 µg/kg and a maximum of 42,143 µg/kg. In Benin, total AFB1 + B2 occurred in 30–42.5% of pre-harvest maize samples, with mean levels around 137–140 µg/kg and maxima reaching 2500 µg/kg. In Ethiopia, median FB1 was 193 µg/kg but reached 11,831 µg/kg, illustrating the gap between typical and extreme contamination. In Nigeria, Tanzania, and Zimbabwe, mean and maximum fumonisin levels were 3851 and 14,603 µg/kg for FB1 + FB2, 1361 and 18,184 µg/kg for FB1, and 2383 and 40,000 µg/kg for total fumonisins, respectively, confirming substantial cross-country variability.

Although many of these values exceed established maximum limits, they should be interpreted with caution, as they do not necessarily reflect average or typical contamination levels. However, they highlight the presence of high-risk contamination events and underscore the persistent gap between regulatory standards and field-level realities, largely driven by limited monitoring capacity, inadequate analytical infrastructure, and the dominance of informal supply chains.

An additional challenge relates to the management of contaminated grain that exceeds safety thresholds. In the absence of economically viable and safe alternative uses, such grain may still enter food or feed chains, highlighting the need for valorisation pathways and risk-based redirection strategies to prevent human and animal exposure (Matumba et al. 2016).

Despite these challenges, several promising perspectives for improving mycotoxin management in Africa are emerging. In addition to the core interventions identified, several cross-cutting dimensions emerged from the synthesis as critical determinants of intervention effectiveness, particularly in shaping adoption, scalability, and long-term impact.

### Perspectives for improving and strengthening mycotoxin management in cereals in Africa

The management of mycotoxins in Africa is not only an agricultural and food safety issue, but also a public health and environmental challenge. A One Health perspective, which recognizes the interconnectedness of human, animal, and environmental health, provides an effective preventive framework. Mycotoxin contamination affects cereals consumed by humans and also by livestock, with repercussions on animal health and the safety of derived products such as milk and meat. Moreover, poor agricultural practices and climate variability contribute to fungal proliferation, illustrating the environmental dimension of the problem. Integrating One Health principles into prevention strategies would therefore promote coordinated actions across sectors: developing sustainable farming systems that reduce fungal risks, strengthening surveillance of mycotoxins in both food and feed, and aligning agricultural, health, and trade policies. Such multisectoral collaboration can improve early warning systems, reduce exposure in both humans and animals, and ultimately support food security and public health.

### Predictive models for mycotoxin contamination

Although predictive modeling was not explicitly captured within the inclusion criteria of this scoping review, it is discussed here as a complementary perspective in response to the identified challenges, particularly climate variability and the need for anticipatory risk management. It is therefore not presented as an intervention category identified through the eligibility criteria, but as a forward-looking gap and supportive tool that complements the evidence synthesized from the included studies.

Because climate variability complicates control, predictive models are being developed as early warning tools. The development of predictive models represents a major advance in the proactive management of mycotoxins (Liu et al. 2018). These models used statistical approaches and artificial intelligence tools to analyse a multitude of environmental, agricultural and climatic variables influencing mycotoxin production (Leggieri et al. 2021). Parameters such as relative humidity, temperature, cultivation practices and the genetic characteristics of crops were integrated to predict the risk of contamination (Klem et al. 2007). According to Battilani (2016), these tools enabled to identify critical periods or geographical areas at risk, making it easier to implement targeted preventive measures, such as choosing resistant cereal varieties, adjusting harvesting schedules or applying biological agents. It is important to note that certain key interventions, such as the selection of resistant crop varieties or the application of biocontrol agents like *Aflasafe*, are typically determined prior to the growing season and are therefore not directly influenced by in-season predictive modeling (Bandyopadhyay et al. 2016). In this context, predictive models are better suited to supporting in-season monitoring, post-harvest management decisions, and longer-term planning strategies aimed at anticipating contamination risks. In addition, predictive models can be enhanced by real-time databases from IoT (Internet of Things) sensors deployed in the field, providing up-to-date information on environmental conditions (Aggarwal et al. 2023). Moreover, these models can be adapted to regional specificities, taking into account local ecological and socio-economic diversities (Liu et al. 2018). In resource-limited context such as Africa, these tools are proving particularly useful for guiding interventions, limiting crop losses and protecting public health (Thyparambil et al. 2017). Several initiatives in sub-Saharan Africa have demonstrated the potential for adapting existing models. The APSIM model, for example, originally designed in Australia, has been successfully tested in Kenya to simulate interactions between climatic and cultural factors (Chauhan et al. 2008, 2015; Nji et al. 2022a, b). Similarly, AFLA-maize, developed for maize in Italy, has been adapted to Malawi, where it has been used to effectively anticipate AFB1 levels in crops, proof that these approaches are transposable to other agroecological contexts (Battilani et al. 2013; Nji et al. 2022a, b; Warnatzsch et al. 2020).

However, these models have certain limitations, mainly due to the variability of climatic conditions, which can complicate their implementation and affect their reliability. Each approach presents specific advantages and constraints, requiring varying degrees of calibration to ensure their effectiveness in particular contexts (Nji et al. 2022a, b).

To ensure their relevance in Africa, predictive models ideally require a range of local data, including climatic (temperature, rainfall, humidity), agronomic (crop types, varieties, cultivation practices), post-harvest (drying and storage methods), as well as analytical data from contamination tests (Battilani 2016). However, in many African contexts, the availability and reliability of such data remain limited (Ngum et al. 2022). While certain parameters can be partially captured through satellite imagery or agricultural surveys, others such as crop varieties or post-harvest practices are more difficult to monitor consistently (Hegarty-Craver et al. 2020). In addition, national laboratory systems often face resource and capacity constraints, limiting their ability to provide large-scale, continuous datasets (Misihairabgwi et al. 2019). As a result, the development and application of predictive models require pragmatic approaches that rely on available data, simplified indicators, and progressive data system strengthening to ensure their practical usability.

The choice of model will depend on the availability of these data. Mechanistic approaches, such as AFLA-maize, are suited to systems with detailed data (Battilani et al. 2013), while empirical or hybrid models incorporating artificial intelligence offer interesting flexibility in more complex or less well-documented environments (Schweidtmann et al. 2024). Whichever approach is chosen, a calibration and validation phase on a local scale is essential (Nji et al. 2022a, b), based on several agricultural seasons to ensure the model’s accuracy and reliability.

Once developed, these tools need to be made accessible via concrete solutions: mobile applications, digital dashboards, or warning systems for producers and agricultural technicians. Their adoption requires support in the field, through the training of key players (cooperatives, NGOs, technical services) and raising farmers’ awareness of the use of these forecasts in their daily practices.

Last but not least, the sustainability of these systems requires their integration into regional agricultural policies, as well as institutional and financial support to enable their continuous updating and large-scale deployment. However, their impact ultimately depends on their integration into practical decision-making processes at the farm level.

In this way, predictive models can serve as supportive tools for preventive and risk-informed management of mycotoxin-related hazards, particularly when combined with practical interventions, extension services, and locally adapted mitigation strategies.

#### Integrating a gender-sensitive approach for enhanced mycotoxin management

While gender was not a primary focus of the literature search, it is considered here as a cross-cutting dimension emerging from the identified socio-economic and behavioral constraints affecting mycotoxin management in African cereal systems.

The integration of a gender-sensitive perspective into mycotoxin management is both a scientific and policy necessity. Evidence indicates that exposure levels, biological susceptibility, and socio-economic determinants of mycotoxin-related risks differ between men and women, potentially biasing risk assessments and weakening the effectiveness of mitigation measures when such differences are overlooked (Thenuwara et al. 2024). Women are particularly involved in high-exposure tasks such as drying, sorting, artisanal milling, processing, and food preparation, while also caring for infants and young children, who are especially vulnerable to toxic effects (Gbashi et al. 2019). This dual role simultaneously heightens women’s exposure and makes them pivotal actors in prevention and mitigation strategies at household and community levels (Leslie et al. 2023).

Several studies highlighted the need for targeted interventions to support women, including technical training, improved drying technologies, hermetic storage solutions, and financial mechanisms such as microcredit to strengthen post-harvest practices (Leslie et al. 2023). Biological differences including hormonal, metabolic, and immunological variations further create specific vulnerability windows such as pregnancy and lactation, reinforcing the importance of prioritising protective measures for women of reproductive age (Thenuwara et al. 2024). Comparative studies confirmed that in certain contexts, women are more exposed than men, mainly because of their central role in cereal-related household and artisanal activities (Abia et al. 2013).

At the institutional level, men continue to dominate decision-making and regulatory bodies, shaping standards, resource allocation, and information flows. Addressing this imbalance requires connecting community-based awareness and practices led by women with institutional reforms that promote their inclusion in decision-making structures (Gbashi et al. 2019; Leslie et al. 2023). Embedding a gender-sensitive lens throughout the risk management cycle from research and screening to regulation and enforcement is therefore essential to ensure equity and optimise the effectiveness of strategies.

Beyond gender-related considerations, mycotoxin management interventions can also be interpreted as generating potential co-benefits across human, animal, and environmental health domains, thereby reinforcing the relevance of a One Health perspective (Table 2). These interlinkages are based on the reported effects of the interventions identified in the reviewed studies, such as reductions in mycotoxin contamination, fungal growth, insect damage, moisture-related spoilage, and exposure pathways, and were further supported by complementary One Health literature. However, they should be understood as evidence-informed potential co-benefits rather than outcomes directly measured across all included studies. This interconnectedness underscores the need for adaptive frameworks capable of integrating social equity, food and feed safety, and ecosystem sustainability within a unified mycotoxin management approach.

**Table 2:** Interpretive mapping of mycotoxin management interventions to potential One Health contributions in African cereal systems

Intervention category	Evidence basis from included studies	Supporting evidence for One Health interpretation	Potential One Health contributions
Biocontrol and Bt maize-based control	AFs/FBs reduction; shift towards non-aflatoxigenic *A. flavus* strains; reduced insect-mediated fungal infection.	(Adisasmito et al. 2022); (Nwaji et al. 2022); (Bryden 2012); (Yang et al. 2020); (Rheeder and van der Westhuizen 2024); (Bandyopadhyay et al. 2019); (Ncube et al. 2018); (Senghor et al. 2020)	May reduce dietary exposure, improve cereal-based feed safety, and support ecological control of toxigenic fungal populations.
Pesticide use	Reduced pest damage and potential indirect reduction of fungal infection.	(Beyuo et al. 2024); (Nyarko et al. 2021); (Adeyeye et al. 2022)	May reduce pest-mediated contamination, but residue risks, misuse and environmental effects require control.
Improved drying	Reduced grain moisture and limited post-harvest fungal growth or toxin build-up.	(Neme and Mohammed 2017); (Nyarko et al. 2021);	May preserve safer cereals for food and feed and reduce spoilage/post-harvest losses.
Hermetic and improved storage	Reduced moisture exchange, insect activity, fungal proliferation and post-harvest contamination.	(Mobolade et al. 2019); (Baoua et al. 2016); (Nyarko et al. 2021)	May protect food/feed stocks, reduce losses, and limit reliance on repeated chemical treatments.
Processing	Reduction/removal of contaminated fractions before consumption.	(Karlovsky et al. 2016);	May reduce dietary exposure and improve management of contaminated by-products or rejected fractions.

This table interprets One Health contributions in cereal-based systems, with groundnuts outside the review’s eligibility criteria. Human health reflects reported reductions in mycotoxin contamination and dietary exposure; animal and environmental effects are potential co-benefits, not directly measured outcomes

Recognising that the limitations and variability in intervention effectiveness identified in the reviewed studies are largely driven by structural, socio-economic, and institutional constraints, these challenges were synthesised into an integrated response through the development of the Integrated Adaptive Mycotoxin Management Framework (IAMMF), which combines technical and non-technical dimensions within a unified and adaptive structure.

The main interventions discussed in this review, along with their effectiveness, supporting evidence, implementation contexts, and adoption barriers, are summarised in Table 3. This synthesis provides an integrated overview of what has proven most effective and where challenges persist, thereby directly informing the design of context-sensitive and sustainable management models, including the Integrated Adaptive Mycotoxin Management Framework (IAMMF). The IAMMF was therefore constructed from the recurrent technical and systemic determinants identified across the included studies, rather than from a separate standalone literature review.

**Table 3:** Comparative synthesis of main mycotoxin interventions - effectiveness, evidence base, contextual applications, and barriers to adoption

Intervention	Typical effect range (%)	Number of supporting studies	Crops	Countries	Main adoption barriers
Biocontrol (Aflasafe atoxigenic Aspergillus flavus, Bt maize/insect-resistant GM maize)	60–100% reduction in Afs, 48–74% rduction in FBs	11	Maize	Nigeria, Ghana, Senegal, Gambia, Tanzania, South Africa	Product cost, limited commercialization, regulatory restrictions, public acceptance concerns, climate variability, lack of multi-toxin control.
Improved drying (raised/suspended, solar, hybrid)	40–90% reduction in fungal growth and AFs	4	Maize, sorghum	Kenya, Ghana, Benin, Tanzania	Infrastructure, weather dependence, low awareness
Hermetic storage (metal silos, PICS bags, improved cribs)	50–95% reduction in insect damage and AFs accumulation	7	Maize, sorghum	Nigeria, Ghana, Ethiopia, Senegal	Cost, accessibility, lack of technical know-how
Processing (sorting, dehulling, fermentation, HACCP)	Up to 70–90% reduction in AFs and FBs	8	Maize, millet, sorghum	Malawi, Tanzania, Nigeria	Infrastructure, training, regulation, process variability
Pesticide use	Indirect benefits (reduction of pest/fungal incidence, no direct toxin reduction)	3	Maize, rice	Benin, Kenya	Misuse, lack of training, residue risks

### Towards an integrated adaptive mycotoxin management framework for Africa

The synthesis of the reviewed studies highlights that mycotoxin management in African cereal systems is constrained not only by technical limitations, but also by structural, institutional, and socio-economic factors. The fragmentation of interventions across the value chain, combined with variability in effectiveness and adoption, reveals that isolated strategies are insufficient to achieve sustainable control. Building on this evidence, the Integrated Adaptive Mycotoxin Management Framework (IAMMF) is proposed as an evidence-informed synthesis that integrates the identified interventions, challenges, and enabling factors into a coherent and adaptive management model.

The development of the IAMMF was guided by a structured synthesis of recurrent themes identified across the included studies. Rather than emerging as a standalone conceptual proposition, each component of the framework was a derived from consistent evidence patterns observed across pre- and post-harvest interventions, including technological effectiveness, implementation constraints, institutional gaps, and socio-economic determinants.

**Fig. 2:**
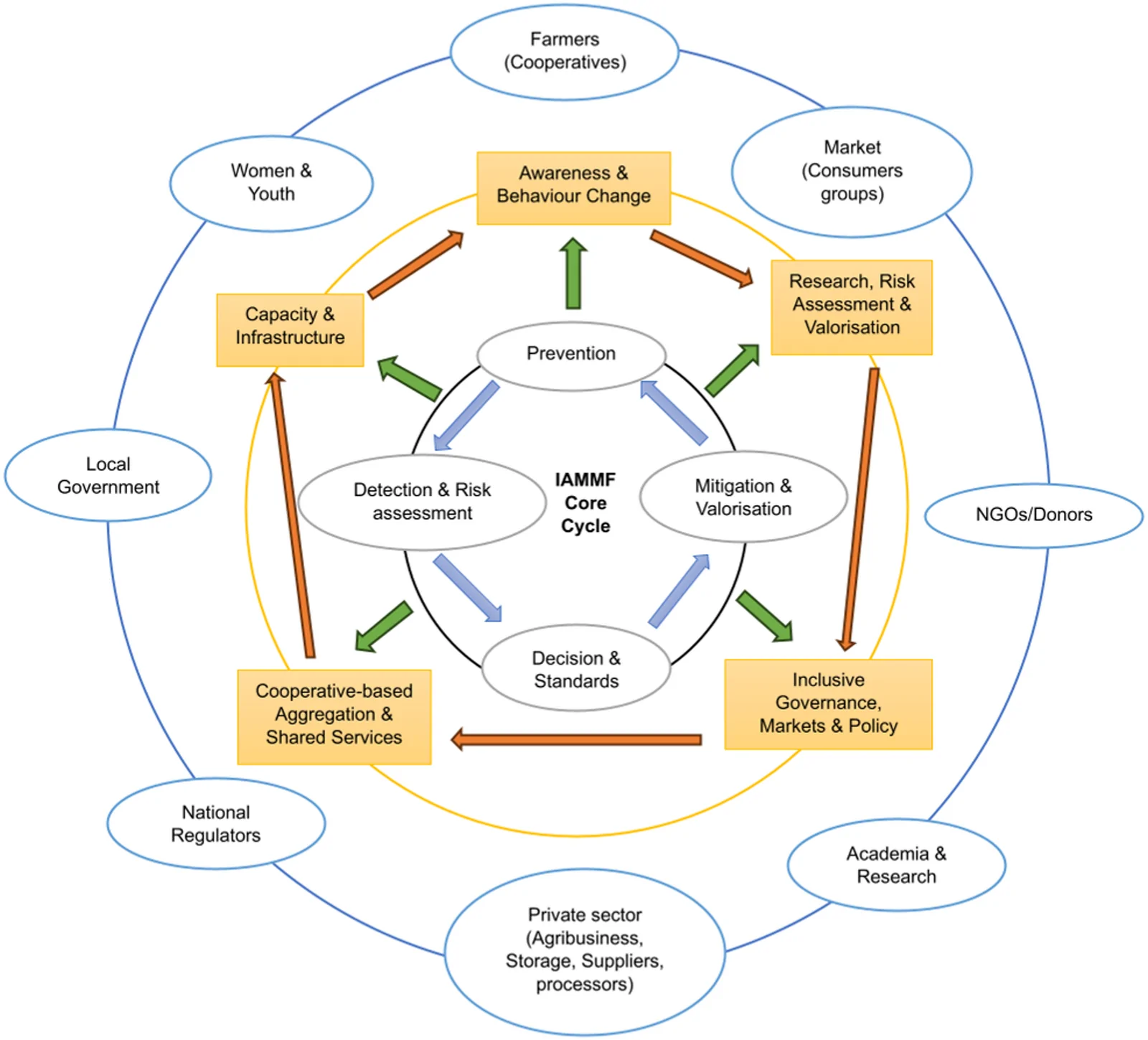
Integrated Adaptive Mycotoxin Management Framework (IAMMF) The IAMMF illustrates the dynamic interactions between prevention, detection, mitigation, and decision-making processes across multiple stakeholder levels in the cereal value chain. It operationalises the One Health approach by integrating human, animal, and environmental health considerations into surveillance, governance, and adaptive management, thereby promoting systemic resilience and sustainable food safety in African contexts

Invisible and odourless, mycotoxins are toxins that farmers, processors, retailers, and consumers often underestimate, despite their severe health and economic consequences. Detection typically relies on laboratory analyses, which remain inaccessible in many African contexts, while markets rarely distinguish between safe and contaminated products. As a result, mycotoxin control in cereals cannot rely on fragmented interventions. It requires an integrated, multi-actors, and adaptive framework that combines prevention, laboratory surveillance, evidence-informed decision-making, valorisation strategies, and inclusive governance (Fig. 2).

The IAMMF illustrates this approach by linking a core cyclic process with five strategic pillars, supported by continuous interactions with key stakeholders across the value chain. The five pillars of the framework are directly informed by the key gaps, constraints, and intervention patterns identified in the reviewed studies. For example, the recurring lack of awareness and unsafe practices informed the “Awareness and Behaviour Change” pillar, while limitations in laboratory capacity and infrastructure underpin the “Capacity and Infrastructure” component. Similarly, the importance of collective action and market access supports the “Cooperative-based Aggregation” pillar, and the identified regulatory fragmentation justifies the “Inclusive Governance” dimension. Finally, the limited availability of intervention-based evidence and the need for continuous adaptation underpin the “Research, Risk Assessment and Valorisation” pillar. The IAMMF is informed by a One Health perspective, recognizing the interconnections between food safety, human health, animal health, and environmental sustainability within African cereal systems.

#### Awareness and behaviour change

This pillar reflects recurring findings across included studies indicating low awareness among farmers and traders regarding mycotoxin risks, leading to suboptimal post-harvest practices such as inadequate drying and poor storage. A persistent driver of contamination is the low level of awareness across the value chain. Farmers frequently adopt risky practices in harvesting, drying, and storage, while traders and processors often ignore the health and economic implications of contaminated grains (James et al. 2007; Waliyar et al. 2015). Even regulators and policymakers may lack updated knowledge to guide their actions (Bandyopadhyay et al. 2016). Systematic and continuous awareness campaigns, tailored to local realities and grounded in participatory approaches, are therefore essential to translate knowledge into sustained behavioural change (Anitha et al. 2019). They should use accessible media such as community radio, visual aids adapted to low literacy, and demonstration plots, while mobilising women’s groups, youth, and community leaders as trusted intermediaries (Leslie et al. 2023).

#### Capacity and infrastructure

This pillar is grounded in evidence highlighting limited access to drying infrastructure, storage technologies, laboratory capacity, and extension services across multiple studies. Strengthening technical and institutional capacity is central to the IAMMF. Extension officers, laboratory staff, and cooperative technicians need targeted training on rapid testing (ELISA kits, strip tests), grain moisture monitoring, and risk assessment (Bandyopadhyay et al. 2016; Waliyar et al. 2015). Affordable infrastructure such as communal drying floors, hermetic storage bags, and solar dryers should be promoted through cooperatives and private service providers (De Groote et al. 2021; Udomkun et al. 2020). Equally important is the development of national and regional laboratories with quality assurance systems that can support surveillance and enforcement (Nji et al. 2022a, b). These capacities form the diagnostic backbone of One Health surveillance, linking agricultural and public health systems through shared data and early detection mechanisms.

#### Cooperative-based aggregation and shared services

Cooperatives play a pivotal role in scaling up prevention and monitoring. By pooling resources, they can invest in communal equipment, coordinate quality-based grain contracts, and facilitate market linkages (Yohannis et al. 2025). They also provide platforms for farmer training, collective bargaining, and monitoring of compliance with food safety standards (Imami et al. 2021). In addition, shared services within cooperatives reduce the burden on individual smallholders while ensuring traceability of grain flows (Paraschou et al. 2025).

### Inclusive governance, markets and policy

This component reflects reported fragmentation in regulatory enforcement, weak market surveillance systems, and inconsistent implementation of mycotoxin standards across countries. Effective mycotoxin management requires governance structures that are adaptive, participatory, and transparent. Multi-stakeholder platforms including women, youth, regulators, and the private sector should oversee the development and revision of standards, integrating scientific evidence and socio-economic realities (Chilaka et al. 2022). Incentive-based enforcement is central: quality premiums, access to finance, and preferential market entry should reward compliance, while transparent penalties should discourage negligence (Grace et al. 2019). Regional harmonisation of standards (through ECOWAS or AU frameworks) could strengthen both intra-African trade and food safety systems (Chilaka et al. 2022).

### Research, risk assessment and valorisation

Continuous research and surveillance are essential for updating policies and guiding interventions. Risk assessments combining exposure studies, toxicology, and socio-economic data provide the evidence base for adaptive standards (Attrey 2017). Research should also explore resistant varieties through breeding and participatory selection, as well as validate traditional mitigation methods such as fermentation and nixtamalisation, an alkaline cooking process traditionally used in maize preparation that can reduce certain mycotoxins by altering their chemical structure (Maris et al. 2025). Moreover, valorisation pathways, referring to the safe redirection of contaminated grain toward alternative uses such as biofuel production or animal feed supplemented with mycotoxin-binding agents, can reduce waste while minimising economic losses for farmers and protecting environmental health (Fumagalli et al. 2021). In the absence of such economically viable and safe alternatives, contaminated grain may still enter food or feed chains, posing continued risks to human and animal health.

## Actor engagement and feedback loops

The IAMMF explicitly recognises the role of diverse actor’s cooperatives, women’s and youth groups, governments, academia, NGOs, donors, private sector, and consumer associations in both implementing practices and providing feedback (Béné 2020; FAO, 2011). Data from surveillance, adoption rates, and market responses flow back into the cycle, enabling iterative learning and adaptive governance (Leeuwis and Aarts 2011).

Overall, the IAMMF represents a structured synthesis of empirical findings from the reviewed studies, translating recurring implementation challenges and intervention outcomes into an integrated, adaptive management framework tailored to African cereal systems.

He provides a systemic, cyclic, and inclusive framework for managing mycotoxins in African cereals. By integrating awareness, capacity building, cooperative action, inclusive governance, and continuous research into a dynamic process, it addresses the limitations of fragmented approaches identified in the reviewed studies. Its adoption offers a pathway to improve coordination across the value chain and to enhance the effectiveness of mycotoxin risk management strategies, with potential benefits for public health, animal productivity, and environmental sustainability. The framework therefore represents a synthesis of empirically observed patterns rather than a purely theoretical construct.

Overall, this scoping review mapped 33 primary studies on mycotoxin management strategies in cereals across Africa and showed that effective mitigation is possible across the cereal value chain. Biocontrol agents, particularly Aflasafe, consistently reduced aflatoxin contamination, while targeted post-harvest interventions such as improved drying, hermetic storage, and processing practices (sorting, dehulling, HACCP-based systems) also lowered mycotoxin levels.

Despite these advances, impact remains uneven. Traditional practices still dominate in many contexts, and adoption of proven technologies is constrained by climatic variability, limited infrastructure, low awareness, gender disparities, informal market dominance, and fragmented regulatory systems. Evidence gaps persist regarding multi-mycotoxin management and scalability across diverse agroecological and socio-economic settings.

The synthesis of the reviewed studies revealed recurring patterns across three key dimensions: (i) the effectiveness and limitations of interventions across the value chain; (ii) structural and contextual challenges affecting their implementation; and (iii) cross-cutting factors such as awareness, capacity, and socio-economic constraints. These interrelated dimensions highlight that mycotoxin risk management effectiveness is not solely determined by technical performance, but by the interaction between interventions and their implementation context.

Building on these interconnected findings, the Integrated Adaptive Mycotoxin Management Framework (IAMMF) is proposed as an evidence-informed synthesis that integrates technical, institutional, and socio-economic dimensions within a unified and adaptive structure. By linking prevention, monitoring, governance, and capacity building, the framework provides a coherent foundation for designing context-sensitive and coordinated strategies to reduce mycotoxin risks in African cereal systems.

## Supplementary information

Below is the link to the electronic supplementary material.

Supplementary File 1 (DOCX 44.3 KB)

## Data Availability

All data generated or analysed during this review are included in this published article.

## References

[CR1] Abia WA, Warth B, Sulyok M, Krska R, Tchana A, Njobeh PB, Turner PC, Kouanfack C, Eyongetah M, Dutton M, Moundipa PF (2013) Bio-monitoring of mycotoxin exposure in Cameroon using a urinary multi-biomarker approach. Food Chem Toxicol 62:927–934. 10.1016/j.fct.2013.10.003 24128729

[CR2] Achi OK, Asamudo NU (2019) Cereal-Based Fermented Foods of Africa as Functional Foods. Ref Ser Phytochemistry 2(4):1527–1558. 10.1007/978-3-319-78030-6_31

[CR3] Ademola O, Saha Turna N, Liverpool-Tasie LSO, Obadina A, Wu F (2021) Mycotoxin reduction through lactic acid fermentation: evidence from commercial ogi processors in southwest Nigeria. Food Control 121(September 2020):107620. 10.1016/j.foodcont.2020.107620

[CR4] Adeyeye SAO, Ashaolu TJ, Idowu-Adebayo F (2022) Mycotoxins: Food Safety, Consumer Health and Africa’s Food Security. Polycycl Aromat Compd 42(8):5779–5795. 10.1080/10406638.2021.1957952

[CR5] Adisasmito WB, Almuhairi S, Behravesh CB, Bilivogui P, Bukachi SA, Casas N, Becerra NC, Charron DF, Chaudhary A, Ciacci Zanella JR, Cunningham AA, Dar O, Debnath N, Dungu B, Farag E, Gao GF, Hayman DTS, Khaitsa M, Koopmans MPG, Cediel Becerra N, Machalaba C, Mackenzie JS, Markotter W, Mettenleiter TC, Morand S, Smolenskiy V, Zhou L (2022) One health: a new definition for a sustainable and healthy future. PLoS Pathog 18(6):2020–2023. 10.1371/journal.ppat.1010537 PMC9223325 35737670

[CR6] African Union Commission (2022) Guide to the African Union

[CR7] Agbetiameh D, Ortega-Beltran A, Awuah RT, Atehnkeng J, Elzein A, Cotty PJ, Bandyopadhyay R (2020) Field efficacy of two atoxigenic biocontrol products for mitigation of aflatoxin contamination in maize and groundnut in Ghana. Biol Control 150(May):104351. 10.1016/j.biocontrol.2020.104351 PMC7457722 33144821

[CR8] Aggarwal M, Sahoo P, Saha S, Das P (2023) Machine learning-mediated ultrasensitive detection of citrinin and associated mycotoxins in real food samples discerned from a photoluminescent carbon dot barcode array. J Agric Food Chem 71(34):12849–12858. 10.1021/acs.jafc.3c04846 37584518

[CR9] Ahouangninou C, Martin T, Edorh P, Bio-Bangana S, Samuel O, St-Laurent L, Dion S, Fayomi B (2012) Characterization of Health and Environmental Risks of Pesticide Use in Market-Gardening in the Rural City of Tori-Bossito in Benin, West Africa. J Environ Prot 03(03):241–248. 10.4236/jep.2012.33030

[CR10] Akanle PhD O, Adesina PhD JO, Ademuson AO (2017) Gender paradoxes and agricultural monopoly in Nigeria: implications for policy and food (in)security in Africa. Gender & Behaviour 15(3), 9665–9677. https://www.proquest.com/scholarly-journals/gender-paradoxes-agricultural-monopoly-nigeria/docview/2113736155/se-2?accountid=36937%0Ahttps://media.proquest.com/media/hms/PFT/1/xpBD7?_a=ChgyMDIzMDQxNzAyMjYyNzIyNjo3MTg3ODUSBTY2NjIwGgpPTkVfU0VBUkNIIg0xODEuNj

[CR11] Akowuah JO, Maier D, Opit G, McNeill S, Amstrong P, Campabadal C, Ambrose K, Obeng-Akrofi G (2018) Drying Temperature Effect on Kernel Damage and Viability of Maize Dried in a Solar Biomass Hybrid Dryer. Open J Appl Sci 08(11):506–517. 10.4236/ojapps.2018.811041

[CR12] Anitha S, Tsusaka TW, Njoroge SMC, Kumwenda N, Kachulu L, Maruwo J, MacHinjiri N, Botha R, Msere HW, Masumba J, Tavares A, Heinrich GM, Siambi M, Okori P (2019) Knowledge, attitude and practice of Malawian farmers on pre- and post-harvest crop management to mitigate aflatoxin contamination in groundnut, maize and sorghum-implication for behavioral change. Toxins (Basel). 10.3390/toxins11120716 PMC6950711 31835420

[CR13] Atehnkeng J, Ojiambo PS, Cotty PJ, Bandyopadhyay R (2014) Field efficacy of a mixture of atoxigenic Aspergillus flavus Link: FR vegetative compatibility groups in preventing aflatoxin contamination in maize (Zea mays L). Biol Control 72:62–70. 10.1016/j.biocontrol.2014.02.009

[CR14] Atehnkeng J, Ojiambo PS, Ortega-Beltran A, Augusto J, Cotty PJ, Bandyopadhyay R (2022) Impact of frequency of application on the long-term efficacy of the biocontrol product Aflasafe in reducing aflatoxin contamination in maize. Front Microbiol 13(November):1–14. 10.3389/fmicb.2022.1049013 PMC9732863 36504767

[CR15] Attrey DP (2017) Role of risk analysis and risk communication in food safety management. Food Safety in the 21st Century: Public Health Perspective, (2015), 53–68. 10.1016/B978-0-12-801773-9.00005-4

[CR16] Augusto J, Atehnkeng J, Ortega-Beltran A, Cotty PJ, Bandyopadhyay R (2024) Keeping toxigenic *Aspergillus* section Flavi and aflatoxin contamination at bay by deploying atoxigenic-based biocontrol products during production of groundnut and maize in Mozambique. Front Microbiol. 10.3389/fmicb.2024.1501924 PMC11615066 39633814

[CR17] Bandyopadhyay R, Akande A, Mutegi C, Atehnkeng J, Kaptoge L, Senghor AL (2016) Biological control of aflatoxins in Africa: current status and potential challenges in the face of climate change Abstract 9(5):771–789. 10.3920/WMJ2016.2130

[CR18] Bandyopadhyay R, Atehnkeng J, Ortega-Beltran A, Akande A, Falade TDO, Cotty PJ (2019) Ground-Truthing Efficacy of Biological Control for Aflatoxin Mitigation in Farmers’ Fields in Nigeria: From Field Trials to Commercial Usage, a 10-Year Study. Front Microbiol 10(November):1–18. 10.3389/fmicb.2019.02528 PMC6882503 31824438

[CR19] Baoua IB, Amadou L, Bakoye ON, Abdoulaye O, Baributsa D, Murdock LL (2016) Maize quality in markets in four West African countries. J Stored Prod Res 69:26–30. 10.1016/j.jspr.2016.05.005

[CR20] Battilani P (2016) Recent advances in modeling the risk of mycotoxin contamination in crops. Curr Opin Food Sci 11:10–15. 10.1016/j.cofs.2016.08.009

[CR21] Battilani P, Camardo Leggieri M, Rossi V, Giorni P (2013) AFLA-maize, a mechanistic model for Aspergillus flavus infection and aflatoxin B1 contamination in maize. Comput Electron Agric 94:38–46. 10.1016/j.compag.2013.03.005

[CR22] Béné C (2020) Resilience of local food systems and links to food security – A review of some important concepts in the context of COVID-19 and other shocks. Food Secur 12(4):805–822. https://link.springer.com/article/10.1007/s12571-020-01076-1 10.1007/s12571-020-01076-1 PMC7351643 32837646

[CR23] Beyuo J, Sackey LNA, Yeboah C, Kayoung PY, Koudadje D (2024) The implications of pesticide residue in food crops on human health: a critical review. Discov Agric. 10.1007/s44279-024-00141-z

[CR24] Bonkoungou S, Dagno K, Basso A, Ekanao T, Atehnkeng J, Agbetiameh D, Neya A, Toure M, Tiendrebeogo A, Konate M, Outani B, Konlambigue M, Callicott KA, Cotty PJ, Dieng I, Falade TDO, Bandyopadhyay R, Ortega-Beltran A (2024) Mitigation of aflatoxin contamination of maize, groundnut, and sorghum by commercial biocontrol products in farmers’ fields across Burkina Faso, Mali, Niger, and Togo. CABI Agric Bioscience 5(1):1–21. 10.1186/s43170-024-00313-3 PMC11554699 39539746

[CR25] Bryden WL (2012) Mycotoxin contamination of the feed supply chain: Implications for animal productivity and feed security. Anim Feed Sci Technol 173(1–2):134–158. 10.1016/j.anifeedsci.2011.12.014

[CR26] Cervini C, Abegaz B, Mohammed A, Elias R, Medina A, Gebre K, Verheecke-Vaessen C (2023) Assessment of agricultural practices by Ethiopian women farmers: existence of gender disparities in access to mycotoxins training. World Mycotoxin J 16(3):227–238. 10.3920/WMJ2022.2827

[CR27] Chauhan YS, Wright GC, Rachaputi NC (2008) Modelling climatic risks of aflatoxin contamination in maize. Aust J Exp Agric 48(3):358–366. 10.1071/EA06101

[CR28] Chauhan Y, Tatnell J, Krosch S, Karanja J, Gnonlonfin B, Wanjuki I, Wainaina J, Harvey J (2015) An improved simulation model to predict pre-harvest aflatoxin risk in maize. Field Crops Res 178:91–99. 10.1016/j.fcr.2015.03.024

[CR29] Chilaka CA, Mally A (2020) Mycotoxin occurrence, exposure and health implications in infants and young children in sub-saharan africa: a review. Foods 9(11). 10.3390/foods9111585 PMC7693847 33139646

[CR30] Chilaka CA, Obidiegwu JE, Chilaka AC, Atanda OO, Mally A (2022) Mycotoxin Regulatory Status in Africa: A Decade of Weak Institutional Efforts. Toxins 14(7):1–20. 10.3390/toxins14070442 PMC9321388 35878180

[CR31] Chulze SN (2010) Strategies to reduce mycotoxin levels in maize during storage: A review. Food Addit Contaminants - Part A 27(5):651–657. 10.1080/19440040903573032 20349375

[CR32] Danso JK, Osekre EA, Opit GP, Arthur FH, Campbell JF, Mbata G, Manu N, Armstrong P, McNeill SG (2019) Impact of storage structures on moisture content, insect pests and mycotoxin levels of maize in Ghana. J Stored Prod Res 81:114–120. 10.1016/j.jspr.2018.11.012

[CR33] De Groote H, Githinji PG, Munya BG, Ricker-Gilbert JE (2021) Economics of open-air sun drying in the maize value chain of Kenya. J Agric Food Res 5:100185. 10.1016/j.jafr.2021.100185

[CR34] Dodds B, In S, Science A (2020) Advances in postharvest management of cereals and grains. In Advances in postharvest management of cereals and grains. 10.1201/9781003047988

[CR35] EFSA (2017) Risks to human and animal health related to the presence of deoxynivalenol and its acetylated and modified forms in food and feed. EFSA J 15(9). 10.2903/j.efsa.2017.4718 PMC7010102 32625635

[CR36] Egwim E, Amanabo M, Y. A., B. M (2013) Nigerian indigenous fermented foods: processes and prospects. Intech 11(tourism):13. https://www.intechopen.com/books/advanced-biometric-technologies/liveness-detection-in-biometrics

[CR37] El-shal A, Morsy H (2020) Explaining firm-level gender productivity differential in Africa 1:1–51

[CR38] Eskola M, Kos G, Elliott CT, Hajšlová J, Mayar S, Krska R (2020) Worldwide contamination of food-crops with mycotoxins: Validity of the widely cited ‘FAO estimate’ of 25%. Crit Rev Food Sci Nutr 60(16):2773–2789. 10.1080/10408398.2019.1658570 31478403

[CR39] Ezekiel CN, Abia WA, Ogara IM, Sulyok M, Warth B, Krska R (2015) Fate of mycotoxins in two popular traditional cereal-based beverages (kunu-zaki and pito) from rural Nigeria. Lwt 60(1):137–141. 10.1016/j.lwt.2014.08.018

[CR40] Ezekiel CN, Ayeni KI, Misihairabgwi JM, Somorin YM, Chibuzor-Onyema IE, Oyedele OA, Abia WA, Sulyok M, Shephard GS, Krska R (2018) Traditionally Processed Beverages in Africa: A Review of the Mycotoxin Occurrence Patterns and Exposure Assessment. Compr Rev Food Sci Food Saf 17(2):334–351. 10.1111/1541-4337.12329 33350081

[CR41] Fandohan P, Zoumenou D, Hounhouigan DJ, Marasas WFO, Wingfield MJ, Hell K (2005) Fate of aflatoxins and fumonisins during the processing of maize into food products in Benin. Int J Food Microbiol 98(3):249–259. 10.1016/j.ijfoodmicro.2004.07.007 15698686

[CR42] FAO STAT (2022) Production of cereals in the world from 2012–2022. 7823–7830. https://www.fao.org/faostat/fr/#data/QCL

[CR43] FAO (2011) The state of food and agriculture 2010–2011. https://www.fao.org/4/i2050e/i2050e00.htm

[CR44] Fapohunda SO, Adewunmi AA (2019) Climate change and mycotoxins. Croatian J Food Sci Technol 11(2):283–290. 10.17508/cjfst.2019.11.2.09

[CR45] Fumagalli F, Ottoboni M, Pinotti L, Cheli F (2021) Integrated mycotoxin management system in the feed supply chain: innovative approaches. Toxins (Basel). 10.3390/toxins13080572 PMC8402322 34437443

[CR46] Fundikira S, Kimanya M, Suleiman R, Boevre M, De, Tesfamariam K, Saeger S, De (2025) Effect of Aflasafe TZ01 ^®^ on aflatoxin reduction and emerging challenges with fusarium mycotoxins in maize from rural Tanzania 1–15 10.3390/toxins17080419 PMC12389803 40864095

[CR47] Gbadegesin LA, Ayeni EA, Tettey CK, Uyanga VA, Aluko OO, Ahiakpa JK, Okoye CO, Mbadianya JI, Adekoya MA, Aminu RO, Oyawole FP, Odufuwa P (2022) GMOs in Africa: status, adoption and public acceptance. Food Control 141(June):109193. 10.1016/j.foodcont.2022.109193

[CR48] Gbashi S, Edwin Madala N, De Saeger S, De Boevre M, Adekoya I, Adebo A, O., Njobeh B, P (2019) The socio-economic impact of mycotoxin contamination in africa. mycotoxins - impact manage strategies 3–22. 10.5772/intechopen.79328

[CR49] Grace D, Bonfoh B, Barbara H, Maurer JJ, Tech V, States U (2019) Editorial: Food safety in low- and middle-income countries

[CR50] Grote U, Fasse A, Nguyen TT, Erenstein O (2021) Food Security and the Dynamics of Wheat and Maize Value Chains in Africa and Asia. Front Sustainable Food Syst 4(February):1–17. 10.3389/fsufs.2020.617009

[CR51] Gueye M, Goergen G, Ndiaye S, Asiedu E, Wathelet J, Lognay G, Seck D (2013) Efficiency of Traditional Maize Storage and Control Methods in Rural Grain Granaries: a Case Study from Senegal. Tropicultura 31(2):129–136

[CR52] Gueye PS, Labou B, Diatte M, Diarra K (2020) La mauvaise pratique phytosanitaire, principale source de contamination du chou au Sénégal. Int J Biol Chem Sci 14(2):539–554. 10.4314/ijbcs.v14i2.19

[CR53] Hegarty-Craver M, Polly J, O’Neil M, Ujeneza N, Rineer J, Beach RH, Lapidus D, Temple DS (2020) Remote crop mapping at scale: Using satellite imagery and UAV-acquired data as ground truth. Remote Sens 12(12):1–15. 10.3390/rs12121984

[CR54] Hove M, De Boevre M, Lachat C, Jacxsens L, Nyanga LK, De Saeger S (2016) Occurrence and risk assessment of mycotoxins in subsistence farmed maize from Zimbabwe. Food Control 69:36–44. 10.1016/j.foodcont.2016.04.038

[CR55] Hussen A (2021) Impact of grain storage method on sorghum grain quality in Ethiopia: A review. J Curr Res Food Sci 2(1):40–45 www.foodresearchjournal.com

[CR56] Imami D, Valentinov V, Skreli E (2021) Food safety and value chain coordination in the context of a transition economy: the role of agricultural cooperatives. 10.5334/ijc.1039

[CR57] James B, Adda C, Cardwell K, Annang D, Hell K, Korie S, Edorh M, Gbeassor F, Nagatey K, Houenou G (2007) Public information campaign on aflatoxin contamination of maize grains in market stores in Benin, Ghana and Togo. Food Addit Contam 24(11):1283–1291. 10.1080/02652030701416558 17852397

[CR58] Johnson AM, Fulton JR, Abdoulaye T, Ayedun B, Widmar NJO, Akande A, Bandyopadhyay R, Manyong V (2018) Aflatoxin awareness and Aflasafe adoption potential of Nigerian smallholder maize farmers. World Mycotoxin J 11(3):437–446. 10.3920/WMJ2018.2345 PMC7797632 33552313

[CR59] Kagot V, Okoth S, De Boevre M, De Saeger S (2019) Biocontrol of *aspergillus* and *fusarium* mycotoxins in Africa: benefits and limitations. Toxins (Basel) 11(2):1–9. 10.3390/toxins11020109 PMC6409615 30781776

[CR60] Kamala A, Ortiz J, Kimanya M, Haesaert G, Donoso S, Tiisekwa B, De Meulenaer B (2015) Multiple mycotoxin co-occurrence in maize grown in three agro-ecological zones of Tanzania. Food Control 54:208–215. 10.1016/j.foodcont.2015.02.002

[CR61] Karlovsky P, Suman M, Berthiller F, De Meester J, Eisenbrand G, Perrin I, Oswald IP, Speijers G, Chiodini A, Recker T, Dussort P (2016) Impact of food processing and detoxification treatments on mycotoxin contamination. Mycotoxin Res 32(4):179–205. 10.1007/s12550-016-0257-7 PMC5063913 27554261

[CR62] Kebede H, Liu X, Jin J, Xing F (2020) Current status of major mycotoxins contamination in food and feed in Africa. Food Control 110:106975. 10.1016/j.foodcont.2019.106975

[CR63] Khodaei D, Javanmardi F, Khaneghah AM (2021) The global overview of the occurrence of mycotoxins in cereals: a three-year survey. Curr Opin Food Sci 39:36–42. 10.1016/j.cofs.2020.12.012

[CR64] Kimanya ME (2015) The health impacts of mycotoxins in the eastern Africa region. Curr Opin Food Sci 6:7–11. 10.1016/j.cofs.2015.11.005

[CR65] Klem K, Váňová M, Hajšlová J, Lancová K, Sehnalová M (2007) A neural network model for prediction of deoxynivalenol content in wheat grain based on weather data and preceding crop. Plant Soil Environ 53(10):421–429. 10.17221/2200-pse

[CR66] Knox J, Hess T, Daccache A, Wheeler T (2012) Climate change impacts on crop productivity in Africa and South Asia. Environ Res Lett. 10.1088/1748-9326/7/3/034032

[CR67] Koumassa OAB, Ouétchéhou R, Hounsou M, Zannou O, Dabadé DS (2025) Factors influencing street ‑ vended foods quality and safety in developing countries: a review. Discover Food. 10.1007/s44187-025-00286-w

[CR68] Laskowski W, Górska-Warsewicz H, Rejman K, Czeczotko M, Zwolińska J (2019) How important are cereals and cereal products in the average Polish diet? Nutrients. 10.3390/nu11030679 PMC6470554 30901972

[CR69] Leeuwis C, Aarts N (2011) Rethinking communication in innovation processes: creating space for change in complex systems. J Agric Educ Ext 17(1):21–36. 10.1080/1389224X.2011.536344

[CR70] Leggieri MC, Mazzoni M, Battilani P (2021) Machine learning for predicting mycotoxin occurrence in maize. Front Microbiol 12:1–10. 10.3389/fmicb.2021.661132 PMC8062859 33897675

[CR71] Lehmane H, Ba R, Dah-Nouvlessounon D, Sina H, Roko G, Bade FT, Socohou A, Adjanohoun A, Baba-Moussa L (2022) Status of Techniques Used to Control Moulds in Maize Storage in Africa. Agricultural Sci 13(01):49–64. 10.4236/as.2022.131005

[CR72] Leslie JF, Morris JB, Gurung JK, Harvey JJW, Ayalew A, Baker R, Zhang G (2023) Mycotoxin communications: Managing messages for different audiences. Front Sustainable Food Syst 6. 10.3389/fsufs.2022.1095256

[CR73] Liu C, Manstretta V, Rossi V, Van Der Fels-Klerx HJ (2018) Comparison of three modelling approaches for predicting deoxynivalenol contamination in winter wheat. Toxins (Basel) 10(7):1–15. 10.3390/toxins10070267 PMC6071054 30004414

[CR74] Liu L, Xie M, Wei D (2022) Biological detoxification of mycotoxins: current status and future advances. Int J Mol Sci. 10.3390/ijms23031064 PMC8835436 35162993

[CR75] Magembe KS, Mwatawala MW, Mamiro DP (2016) Mycotoxin contamination in stored maize and groundnuts based on storage practices and conditions in subhumid tropical Africa: The case of kilosa district, Tanzania. J Food Prot 79(12):2160–2166. 10.4315/0362-028X.JFP-15-550 28221960

[CR76] Magombeyi MS, Taigbenu AE (2008) Crop yield risk analysis and mitigation of smallholder farmers at quaternary catchment level: case study of B72A in Olifants river basin, South Africa. Physics and Chemistry of the Earth, Parts A/B/C 33:744–756. 10.1016/j.pce.2008.06.050

[CR77] Mahai S, Jamala G, Mada D, Medugu I (2015) Assessment of sorghum storage methods in Madagali and Ganye areas of Adamawa State, Nigeria. Int J Engr Sci 10(4):1–6

[CR78] Mahuku G, Mauro A, Pallangyo B, Nsami E, Boni SB, Koyano E, Mponda O, Ortega-Beltran A, Atehnkeng J, Aquiline F, Samuel R, Njela J, Cotty PJ, Bandyopadhyay R (2023) Atoxigenic-based technology for biocontrol of aflatoxin in maize and groundnuts for Tanzania. World Mycotoxin J 16(1):59–73. 10.3920/WMJ2021.2758

[CR79] Mapfeka RF, Mandumbu R, Zengeza T, Kamota A, Masamha B, Marongwe FD, Mutsamba-Magwaza EF, Nyakudya E, Nyamadzawo G (2019) Post-harvest cereal structures and climate change resilience in rural Zimbabwe: A review. Int J Postharvest Technol Innov 6(4):239–256. 10.1504/IJPTI.2019.106460

[CR80] Maris E, Ndlangamandla P, Adelusi OA, Akinmoladun OF, Odukoya JO, Fagbohun RT, Oyeyinka SA, Sekhejane P, Pero-Gascon R, De Boevre M, Croubels S, Njobeh PB, De Saeger S (2025) Nixtamalization of Maize to Reduce Mycotoxin Exposure: A Human Biomonitoring Intervention Study in Soweto, South Africa. Toxins 17(11):1–20. 10.3390/toxins17110527 PMC12656582 41295842

[CR81] Matavel C, Hoffmann H, Rybak C, Sieber S, Müller K, Brüntrup M, Salavessa J (2021) Passive solar dryers as sustainable alternatives for drying agricultural produce in sub-Saharan Africa: advances and challenges. Discov Sustain. 10.1007/s43621-021-00049-4

[CR82] Matumba L, Van Poucke C, Njumbe Ediage E, Jacobs B, De Saeger S (2015) Effectiveness of hand sorting, flotation/washing, dehulling and combinations thereof on the decontamination of mycotoxin-contaminated white maize. Food Addit Contam Part A Chem Anal Control Expo Risk Assess 32(6):960–969. 10.1080/19440049.2015.1029535 25785488

[CR83] Matumba L, Monjerezi M, Kankwamba H, Njoroge SMC, Ndilowe P, Kabuli H, Kambewa D, Njapau H (2016) Knowledge, attitude, and practices concerning presence of molds in foods among members of the general public in Malawi 27–36. 10.1007/s12550-015-0237-3 26711441

[CR84] Matumba L, Van Poucke C, Njumbe Ediage E, De Saeger S (2017) Keeping mycotoxins away from the food: does the existence of regulations have any impact in Africa? Crit Rev Food Sci Nutr 57(8):1584–1592. 10.1080/10408398.2014.993021 25898143

[CR85] Mboya RM, Kolanisi U (2014) Subsistence Farmers’ Mycotoxin Contamination Awareness in the SADC Region: Implications on Millennium Development Goal 1, 4 and 6. J Hum Ecol 46(1):21–31. 10.1080/09709274.2014.11906702

[CR86] Medina A, Rodriguez A, Magan N (2014) Effect of climate change on *Aspergillus flavus* and aflatoxin B1 production. Front Microbiol 5(JULY):1–7. 10.3389/fmicb.2014.00348 PMC4106010 25101060

[CR87] Midega CAO, Murage AW, Pittchar JO, Khan ZR (2016) Managing storage pests of maize: Farmers’ knowledge, perceptions and practices in western Kenya. Crop Prot 90:142–149. 10.1016/j.cropro.2016.08.033

[CR88] Misihairabgwi JM, Ezekiel CN, Sulyok M, Shephard GS, Krska R (2019) Mycotoxin contamination of foods in Southern Africa: A 10-year review (2007–2016). Crit Rev Food Sci Nutr 59(1):43–58. 10.1080/10408398.2017.1357003 28799776

[CR89] Mobolade AJ, Bunindro N, Sahoo D, Rajashekar Y (2019) Traditional methods of food grains preservation and storage in Nigeria and India. Annals Agricultural Sci 64(2):196–205. 10.1016/j.aoas.2019.12.003

[CR90] Molnár K, Rácz C, Dövényi-Nagy T, Bakó K, Pusztahelyi T, Kovács S, Adácsi C, Pócsi I, Dobos A (2023) The effect of environmental factors on mould counts and AFB1 toxin production by *Aspergillus flavus* in maize. Toxins (Basel) 15(3):1–18. 10.3390/toxins15030227 PMC10055717 36977118

[CR91] Mousavi Khaneghah A, Fakhri Y, Gahruie HH, Niakousari M, Sant’Ana AS (2019) Mycotoxins in cereal-based products during 24 years (1983–2017): A global systematic review. Trends Food Sci Technol 91(June):95–105. 10.1016/j.tifs.2019.06.007

[CR92] Murage AW, Pittchar JO, Midega CAO, Onyango CO, Khan ZR (2015) Gender specific perceptions and adoption of the climate-smart push-pull technology in eastern Africa. Crop Prot 76:83–91. 10.1016/j.cropro.2015.06.014

[CR93] Muthomi JW, Mureithi BK, Chemining GN, Gathumbi JK, Mutit EW (2012) Aspergillus species and Aflatoxin b 1 in soil, maize grain and flour samples from semi-arid and humid regions of Kenya. Int J 2(January):22–34. https://www.cabidigitallibrary.org/doi/full/

[CR94] Mutungi C, Muthoni F, Bekunda M, Gaspar A, Kabula E, Abass A (2019) Physical quality of maize grain harvested and stored by smallholder farmers in the Northern highlands of Tanzania: Effects of harvesting and pre-storage handling practices in two marginally contrasting agro-locations. J Stored Prod Res 84:101517. 10.1016/j.jspr.2019.101517

[CR95] Ncube E, Flett BC, Van den Berg J, Erasmus A, Viljoen A (2018) Fusarium ear rot and fumonisins in maize kernels when comparing a Bt hybrid with its non-Bt isohybrid and under conventional insecticide control of Busseola fusca infestations. Crop Prot 110:183–190. 10.1016/j.cropro.2017.09.015

[CR96] Ndemera M, De Boevre M, De Saeger S (2020) Mycotoxin management in a developing country context: A critical review of strategies aimed at decreasing dietary exposure to mycotoxins in Zimbabwe. Crit Rev Food Sci Nutr 60(4):529–540. 10.1080/10408398.2018.1543252 30501517

[CR97] Neme K, Mohammed A (2017) Mycotoxin occurrence in grains and the role of postharvest management as a mitigation strategies. A review. Food Control 78:412–425. 10.1016/j.foodcont.2017.03.012

[CR98] Ngum NQ, Babalola OO, Ekwomadu TI, Nleya N, Mulunda M (2022) Six Main Contributing Factors to High Levels of Mycotoxin Contamination in African Foods. Toxins 14(5):1–26. 10.3390/toxins14050318 PMC9146326 35622564

[CR99] Ngure FM, Makule E, Mgongo W, Phillips E, Kassim N, Stoltzfus R, Nelson R (2024) Processing complementary foods to reduce mycotoxins in a medium scale Tanzanian mill: A hazard analysis critical control point (HACCP) approach. Food Control 162(March):110463. 10.1016/j.foodcont.2024.110463 PMC11064123 39092408

[CR100] Nji QN, Babalola OO, Mwanza M (2022a) Aflatoxins in maize: can their occurrence be effectively managed in Africa in the face of climate change and food insecurity? Toxins (Basel). 10.3390/toxins14080574 PMC9412283 36006236

[CR101] Nji QN, Babalola OO, Nleya N, Mwanza M (2022b) Underreported Human Exposure to Mycotoxins: The Case of South Africa. Foods 11(17):1–19. 10.3390/foods11172714 PMC9455755 36076897

[CR102] Nwaigwe KN (2019) An Overview of Cereal Grain Storage Techniques and Prospects in Africa. Int J Bioeng Biotechnol 4(2):19–25. http://www.openscienceonline.com/journal/ijbb

[CR103] Nwaji AR, Arieri O, Anyang AS, Nguedia K, Abiade EB, Forcados GE, Oladipo OO, Makama S, Elisha IL, Ozele N, Gotep JG (2022) Natural toxins and One Health: a review. Science in One Health. 10.1016/j.soh.2023.100013 PMC11262277 39076609

[CR104] Nyarko SK, Akyereko YG, Akowuah JO, Wireko-Manu FD (2021) Comparative studies on grain quality and pesticide residues in maize stored in hermetic and polypropylene storage bags. Agric (Switzerland) 11(8):1–10. 10.3390/agriculture11080772

[CR105] Okparavero NF, Otitodun GO, Rukayat Q, Jimoh OM, Ishola TD, Okunlade AF, Haruna PB, Isaac AY, Akande ET (2024) Effective Storage Structures for Preservation of Stored Grains in Nigeria: A Review. Ceylon J Sci 53(1):139–147. 10.4038/cjs.v53i1.8194

[CR106] Opoku B, Osekre EA, Opit G, Bosomtwe A, Bingham GV (2023) Evaluation of Hermetic Storage Bags for the Preservation of Yellow Maize in Poultry Farms in Dormaa Ahenkro. Ghana Insects 14(2):1–12. 10.3390/insects14020141 PMC9964676 36835710

[CR107] Paraschou M, Sergaki P, Kalogeras N, Nastis SA, Staboulis C (2025) Agricultural Cooperatives: Roadblocks to Achieving Sustainability. Sustain (Switzerland) 17(17):1–15. 10.3390/su17178012

[CR108] Rheeder JP, van der Westhuizen L (2024) Fusarium and fumonisin in GM maize grown by small-scale farmers in KwaZulu-Natal, South Africa. South Afr J Sci 120(1–2):1–5. 10.17159/sajs.2024/15905

[CR109] Roudier P, Sultan B, Quirion P, Berg A (2011) The impact of future climate change on West African crop yields: What does the recent literature say? Glob Environ Change 21(3):1073–1083. 10.1016/j.gloenvcha.2011.04.007

[CR110] Schweidtmann AM, Zhang D, von Stosch M (2024) A review and perspective on hybrid modeling methodologies. Digital Chemical Engineering. 10.1016/j.dche.2023.100136

[CR111] Senghor LA, Ortega-Beltran A, Atehnkeng J, Callicott KA, Cotty PJ, Bandyopadhyay R (2020) The atoxigenic biocontrol product Aflasafe SN01 is a valuable tool to mitigate aflatoxin contamination of both maize and groundnut cultivated in Senegal. Plant Dis 104(2):510–520. 10.1094/PDIS-03-19-0575-RE 31790640

[CR112] Senghor AL, Ortega-Beltran A, Atehnkeng J, Jarju P, Cotty PJ, Bandyopadhyay R (2021) Aflasafe SN01 is the first biocontrol product approved for aflatoxin mitigation in two nations, Senegal and the Gambia. Plant Dis 105(5):1461–1473. 10.1094/PDIS-09-20-1899-RE 33332161

[CR113] Suleiman RA, Rosentrater KA, Chove B (2017) Postharvest practices and mycotoxins of maize in three agro-ecological zones in Tanzania. 2017 ASABE Annual Int Meeting 8(July):73–84. 10.13031/aim.201700634

[CR114] Sultan B, Roudier P, Quirion P, Alhassane A, Muller B, Dingkuhn M, Ciais P, Guimberteau M, Traore S, Baron C (2013) Assessing climate change impacts on sorghum and millet yields in the Sudanian and Sahelian savannas of West Africa. Environ Res Lett. 10.1088/1748-9326/8/1/014040

[CR115] Tanumihardjo SA, McCulley L, Roh R, Lopez-Ridaura S, Palacios-Rojas N, Gunaratna NS (2020) Maize agro-food systems to ensure food and nutrition security in reference to the Sustainable Development Goals. Glob Food Secur 25:100327. 10.1016/j.gfs.2019.100327

[CR116] Thenuwara G, Javed B, Singh B, Byrne HJ, Tian F (2024) Sex- and gender-specific considerations in mycotoxin screening: assessing differential exposure, health impacts, and mitigation strategies. Microbiol Res 15(4):2455–2492. 10.3390/microbiolres15040165

[CR117] Thyparambil AA, Bazin I, Guiseppi-Elie A (2017) Molecular modeling and simulation tools in the development of peptide-based biosensors for mycotoxin detection: Example of ochratoxin. Toxins 9(12):1–24. 10.3390/toxins9120395

[CR118] Toffa J, Loko YLE, Djedatin G, Gbemavo CDSJ, Orobiyi A, Tchakpa C, Ewedje EE, Sabot F (2021) Rice pests in the Republic of Benin: farmers’ perceptions, knowledge and management practices. Pest Manag Sci 77(11):5058–5071. 10.1002/ps.6546 34227252

[CR119] Tohonon AC, Ouétchéhou R, Hounsou M, Zannou O, Dabadé DS (2024) Food hygiene in Sub-Saharan Africa: a focus on catering services. Food Control. 10.1016/j.foodcont.2024.110938

[CR120] Tovide N, Tchobo F, Adeoti K, Noumavo P, Baba-Moussa F, Gandonou C, Toukourou F (2017) Contamination of cereals (*Sorghum bicolor* L. Moench and *Pennisetum glaucum* (L.) R. Br.) during storage: farmer’s perception and management of mold’ contamination risks. Eur Sci J ESJ 13(27):171. 10.19044/esj.2017.v13n27p171

[CR121] Udomkun P, Wiredu AN, Nagle M, Bandyopadhyay R, Müller J, Vanlauwe B (2017) Mycotoxins in Sub-Saharan Africa: present situation, socio-economic impact, awareness, and outlook. Food Control 72:110–122. 10.1016/j.foodcont.2016.07.039

[CR122] Udomkun P, Romuli S, Schock S, Mahayothee B, Sartas M, Wossen T, Njukwe E, Vanlauwe B, Müller J (2020) Review of solar dryers for agricultural products in Asia and Africa: an innovation landscape approach. J Environ Manage 268:110730. 10.1016/j.jenvman.2020.110730 32510451

[CR123] van der Westhuizen L, Shephard GS, Rheeder JP, Burger HM, Gelderblom WCA, Wild CP, Gong YY (2010) Simple intervention method to reduce fumonisin exposure in a subsistence maize-farming community in South Africa. Food Addit Contam Part A Chem Anal Control Expo Risk Assess 27(11):1582–1588. 10.1080/19440049.2010.508050 20835935

[CR124] van Egmond HP, Jonker MA (2003) Current situation on regulations for mycotoxins. Mycotoxins Suppl3:1–15. 10.2520/myco1975.2003.suppl3_1

[CR125] Waliyar F, Osiru M, Ntare BR, Vijay Krishna Kumar K, Sudini H, Traore A, Diarra B (2015) Post-harvest management of aflatoxin contamination in groundnut. World Mycotoxin J 8(2):245–252. 10.3920/WMJ2014.1766

[CR126] Wambui G, Marther W (2018) Women in african agriculture: integrating women into value (175)

[CR127] Warnatzsch EA, Reay DS, Camardo Leggieri M, Battilani P (2020) Climate change impact on aflatoxin contamination risk in Malawi’s maize crops. Front Sustain Food Syst. 10.3389/fsufs.2020.591792

[CR128] Yang C, Song G, Lim W (2020) Effects of mycotoxin-contaminated feed on farm animals. J Hazard Mater. 10.1016/j.jhazmat.2020.122087 32004836

[CR129] Yohannis E, Urugo MM, Teka TA, Getachew P, Tola YB, Forsido SF, Kebede YS, Teferra TF (2025) Aflatoxin Contamination in Agri-Food Systems: A Comprehensive Review of Toxicity, Food Security, Economic Impacts, and Sustainable Mitigation Across the Value Chain. Food Science & Nutrition 13(10):e71104. 10.1002/fsn3.71104 PMC12529245 41111890

