# Supplementary material for: An integrated adaptive framework for mycotoxin management in African cereals: evidence from a scoping review
Source: Mycotoxin Res. 2026 Sep 24;42(4):71. doi: 10.1007/s12550-026-00673-x (PMC13612647; doi:10.1007/s12550-026-00673-x)
Supplement: Supplementary file 1 [file 12550_2026_673_MOESM1_ESM.docx]

**Table S1:** Mycotoxin limits set by various African countries

| Country | Food products | Mycotoxins | Maximum limit (µg/kg) | References |
| --- | --- | --- | --- | --- |
| Algeria | Peanuts, nuts, cereals | AFB1 | 10 | FAO (2004) |
| Benin | Maize | AFB1 | 5 | MAEP (2007) |
|  | All products derived from raw cereals, including processed cereal products and cereals intended for direct human consumption except: cereal-based preparations and baby foods intended for infants and young children; dietetic foods intended for special medical purposes specifically for infants. | OTA | 3 |  |
|  | Cereal-based preparations and baby foods for infants and young children | DON | 200 |  |
|  | Raw maize | ZEN | 200 |  |
|  | Cereals other than maize |  | 100 |  |
|  | Cereals intended for direct human consumption, cereal flour, bran as a final product placed on the market for direct human consumption and germ |  | 75 |  |
|  | Maize for direct human consumption, maize flour, ground maize, maize grits, maize germ and refined maize oil |  | 200 |  |
|  | Raw maize | FBs | 2000 |  |
|  | Corn flour, ground corn, corn grits, corn germ and refined corn oil |  | 1000 |  |
| Ivory Coast | Food products for animals | AFs | 10 | FAO (2004) |
| Egypt | Peanuts and cereals | AFB1 | 5 |  |
|  | Maize | AFs  (B1, B2, G1, G2) | 10 |  |
| Kenya | Peanuts, vegetable oils | AFs  (B1, B2, G1, G2) | 20 |  |
| Malawi | Groundnuts for export | AFB1 | 5 |  |
| Morocco | All human power supplies | AFB1 | 10 |  |
|  | Cereals, vegetable oils | ZEN | 200 |  |
|  | Cereals | OTA | 30 |  |
| Mozambique | Peanuts, peanut milk | AFs  (B1, B2, G1, G2) | 10 |  |
| Nigeria | Foodstuffs | AFB1 | 20 |  |
| Senegal | Animal feed products derived from peanuts | AFB1 | 50 |  |
| South Africa | All human power supplies | AFB1 | 5 |  |
|  |  | AFs  (B1, B2, G1, G2) | 10 |  |
| Sudan | Wheat | OTA | 15 |  |
| Tanzania | Cereals, oilseeds | AFB1 | 5 |  |
|  |  | AFs  (B1, B2, G1, G2) | 10 |  |
| Tunisia | All products | AFB1 | 2 |  |
| Zimbabwe | Peanuts, maize, sorghum | AFB1 | 5 |  |
|  |  | AFG1 | 4 |  |

**Table S2:** Occurrence of mycotoxins in raw maize across African countries

| Country | Commodity | Mycotoxin | Prevalence (%) | Mean (µg/kg) | Median (µg/kg) | Maximum concentration (µg/kg) | References |
| --- | --- | --- | --- | --- | --- | --- | --- |
| Algeria | Raw maize | DON | 43.3 | 632 | NR | 2055 | Mahdjoubi et al. (2020) |
|  |  | ZEN | 23.3 | 109 | NR | 579 |  |
|  |  | FB1 | 96.6 | 14,812 | NR | 42,143 |  |
|  |  | FB2 | 90 | 2789 | NR | 8603 |  |
| Benin |  | AFB1+AFB2 | 30-42.5 | 136.8-139.8 | NR | 2500 | Sétamou et al. (1997) |
| Congo (Democratic Republic) |  | AFs | 100 | 25.0 | NR | 320 | Udomkun et al. (2018) |
| Côte d'Ivoire |  | AFB1 | 100 | NR | NR | 20 | Sangare-Tigori et al. (2006) |
|  |  | FB1 | 100 | 0.9 | NR | 1.5 |  |
|  |  | OTA | 100 | NR | NR | 0.86 |  |
|  |  | ZEN | 100 | NR | NR | 50 |  |
| Egypt |  | AFB1 | 25 | 8.7 | 1.35 | 44.9 | Abdallah et al. (2019) |
|  |  | AFB2 | 10 | 2.2 | 1.7 | 7.0 |  |
| Ethiopia |  | AFB1 | 8 | 9.3 | 4.8 | 513 | Getachew et al. (2018) |
|  |  | DON | 42 | 221 | 217 | 595 |  |
|  |  | ZEN | 96 | 92 | 9 | 1656 |  |
|  |  | FB1 | 70 | 606 | 193 | 11,831 |  |
|  |  | FB2 | 62 | 202 | 57 | 3732 |  |
| Kenya |  | AFs | 45 | 53 | 43 | 480 | Kilonzo et al. (2014) |
| Morocco |  | FB1 | 50 | 1930 | NR | 5960 | Marín et al. (2012) |
| Nigeria |  | AFB1 | 69 | 54 | NR | 315 | Gbashi et al. (2020) |
|  |  | AFs | 69 | 56 | NR | 323 |  |
|  |  | FB1 | 100 | 2033 | NR | 7947 |  |
|  |  | FB2 | 100 | 1525 | NR | 5961 |  |
|  |  | FB1+FB2 | 100 | 3851 | NR | 14,603 |  |
|  |  | OTA | 31 | 8.3 | NR | 54 |  |
|  |  | ZEN | 19 | 3.5 | NR | 33 |  |
| South Africa |  | FB1 | 92 | 456.2 | NR | 2153 | Tebele et al. (2020) |
|  |  | FB3 | 92 | 396.6 | NR | 1864.6 |  |
|  |  | DON | 21 | 119.2 | NR | 182.2 |  |
| Tanzania |  | AFB1 | 50 | 65 | NR | 1081 | Kamala et al. (2015) |
|  |  | FB1 | 73 | 1361 | NR | 18,184 |  |
|  |  | FB2 | 48 | 2921 | NR | 38,217 |  |
|  |  | DON | 63 | 490 | NR | 2196 |  |
|  |  | ZEN | 10 | 729 | NR | 1464 |  |
| Togo |  | AFs | 42 | 38.7 | NR | 275 | Hanvi et al. (2019) |
|  |  | FB1 | 88 | 504.8 | NR | 1838 |  |
|  |  | FB2 | 55 | 176 | NR | 586.4 |  |
| Zimbabwe |  | FB1 | 95 | 242 | 146 | 1106 | Hove et al. (2016) |
|  |  | DON | 24 | 217 | ND | 492 |  |
|  |  | ZEN | 15 | 110 | ND | 369 |  |
|  |  | AFs | 51.2 | 16.8-17.2 | NR | 1369 | Akello et al. (2021) |
|  |  | FBs | 88.9 | 2383 | NR | 40,000 |  |

**Note:** NR = not reported; ND = not detected.
AFB1 = aflatoxin B1; AFB2 = aflatoxin B2; AFG1 = aflatoxin G1; AFG2 = aflatoxin G2;
AFs = total aflatoxins;
FB1 = fumonisin B1; FB2 = fumonisin B2; FB3 = fumonisin B3; FB4 = fumonisin B4;
FBs = total fumonisins;
FB1+FB2 = sum of fumonisin B1 and B2;
DON = deoxynivalenol; ZEN = zearalenone;
OTA = ochratoxin A; OTB = ochratoxin B;
OTA+OTB = sum of ochratoxin A and B.
